# Multidimensional Effects of Telemedicine on Patients With Spinal Cord Injury: Systematic Review and Meta-Analysis of Randomized Controlled Trials

**DOI:** 10.2196/87088

**Published:** 2026-05-06

**Authors:** Wen Zhong, Liyi Huang, Xin Sun, Rui Liu, Lu Wang, Quan Wei

**Affiliations:** 1 Rehabilitation Medicine Center and Institute of Rehabilitation Medicine, West China Hospital, Sichuan University, Wuhou District, No.37 Guoxue Alley, Chengdu, 610041, China, 86 18980606730; 2 Key Laboratory of Rehabilitation Medicine in Sichuan Province, West China Hospital, Sichuan University, Chengdu, 610041, China

**Keywords:** spinal cord injury, telemedicine, meta-analysis, randomized controlled trials, mental health, quality of life, sleep, functional independence, pain

## Abstract

**Background:**

Spinal cord injury (SCI) causes persistent physical and psychological impairments and is associated with reduced quality of life. Telemedicine may improve rehabilitation access and follow-up care, but its effectiveness across multiple outcome domains in SCI remains uncertain.

**Objective:**

This study aimed to evaluate the effects of telemedicine interventions on psychological health, quality of life, sleep, functional independence, and participation, and pain intensity in individuals with SCI.

**Methods:**

We searched PubMed, Web of Science, Embase, Ovid MEDLINE, and Cochrane CENTRAL until 17 February 2026. We included English-language randomized controlled trials (RCTs) of telemedicine interventions in individuals with SCI. Two reviewers independently screened studies, extracted data, and assessed risk of bias using the Risk of Bias 2 (RoB 2; Cochrane) tool. Random-effects meta-analyses used the Hartung-Knapp-Sidik-Jonkman method with restricted maximum likelihood estimation of between-study variance. Effects were summarized as standardized mean differences (SMD) or mean differences (MD) with 95% CIs. For main meta-analyses, 95% prediction intervals were reported when at least 5 studies were available, but not for analyses with fewer than 5 studies or for subgroup meta-analyses. Certainty of evidence was assessed using GRADE (Grading of Recommendations Assessment, Development, and Evaluation).

**Results:**

We included 33 studies (35 reports). Telemedicine improved the World Health Organization Quality of Life-BREF (WHOQOL-BREF) social domain (MD 3.27, 95% CI 0.64 to 5.89; *P*=.03) and sleep quality at 3 months (MD −2.24, 95% CI −3.82 to −0.67; *P*=.04). Depressive symptoms also improved in the >3-≤6 months follow-up subgroup (SMD −0.31, 95% CI −0.57 to −0.04; *P*=.03). Overall effects for depressive symptoms were not significant (SMD −0.11, 95% CI −0.26 to 0.05; prediction interval −0.37 to 0.15; *P*=.16; *I*²=36.3%), while findings for anxiety, other WHOQOL-BREF domains, sleep quality at 1 month, functional outcomes, and pain intensity generally favored telemedicine but did not reach statistical significance. Approximately half of the studies were rated as low risk overall on RoB 2, with most remaining studies rated as having some concerns and a smaller subset rated as high risk. GRADE certainty was high for the >3-≤6-month depressive-symptoms subgroup, moderate for the WHOQOL-BREF social domain, Pittsburgh Sleep Quality Index (PSQI), and Spinal Cord Independence Measure (SCIM), and low for depressive symptoms overall, anxiety, and pain intensity.

**Conclusions:**

Telemedicine may improve selected outcomes in SCI, with the most consistent evidence for social aspects of quality of life, sleep after sustained intervention exposure, and a more favorable effect on depressive symptoms in midterm follow-up subgroup analyses. These results suggest telemedicine as a practical adjunct for extending SCI rehabilitation access and continuity. Further trials should focus on optimizing intervention components, intensity, and patient targeting.

## Introduction

Spinal cord injury (SCI) refers to damage to the spinal cord resulting from various causes, leading to temporary or permanent neurological dysfunction [1,2]. The etiology of SCI can be broadly classified into traumatic and nontraumatic categories. Traumatic SCI is typically caused by external physical impacts that induce acute injury, whereas nontraumatic SCI results from acute or chronic diseases that compromise the structure and function of the spinal cord. In traumatic SCI, both primary injury and subsequent secondary injury profoundly alter spinal cord tissue structure, ultimately leading to irreversible neurological deficits. According to the 2021 Global Burden of Disease study, there were an estimated 15,400,682 prevalent cases of SCI worldwide, with the majority occurring among working-age individuals (20‐69 years) [3]. The global incidence is estimated at 23.77 cases per million population, with traumatic SCI accounting for 26.48 cases per million and nontraumatic SCI for 17.93 cases per million [4]. SCI not only results in severe motor and sensory impairments but is also frequently complicated by pulmonary infections, deep vein thrombosis, pressure ulcers, and joint contractures, imposing devastating effects on patients’ physical health, social participation, and occupational capacity [5]. Consequently, patients with SCI often experience varying degrees of loss of independence and face an increased risk of mortality.

Telemedicine has emerged as one of the most significant innovations in modern health care, offering new opportunities for the long-term management and rehabilitation of patients with SCI. Given the challenges of limited mobility and restricted access to specialized rehabilitation services, telemedicine is particularly well-suited to this population. Telemedicine services can be delivered via telephone, videoconferencing, computer-based platforms, and robotic-assisted audio or video communication. Based on the target of service delivery, telemedicine can be classified into provider-to-provider (health care professionals to other clinicians or caregivers), direct-to-patient (health care professionals to patients), store-and-forward (transfer of health information to recipients), web-based educational interventions, and interactive home monitoring [6]. Evidence suggests that telemedicine may provide substantial benefits in self-management, psychological well-being, complication prevention, pain control, and functional recovery among patients with SCI. However, the heterogeneity in study objectives, intervention modalities, and outcome measures has limited the identification of optimal strategies and robust conclusions, thereby constraining the broader application of telemedicine in SCI care.

Several systematic reviews have attempted to evaluate the effectiveness of telemedicine in SCI. For instance, a meta-analysis published in 2022 demonstrated that telemedicine interventions significantly reduced the incidence of pressure injuries and improved Pressure Ulcer Scale for Healing (PUSH) scores among community-dwelling patients with SCI. Furthermore, network meta-analysis indicated that hybrid comprehensive telemedicine interventions were more effective than partial telemedicine, no telemedicine, or single-modality telemedicine. Nonetheless, as only four randomized controlled trials (RCTs) could be pooled for analyses of healing rates and PUSH scores, the robustness of these findings remains limited [7]. More recently, a meta-analysis published in 2025 reported that telemedicine significantly improved the quality of life and reduced depressive symptoms in patients with SCI. However, the quality-of-life analysis was characterized by high heterogeneity; for depression, although subgroup analyses were performed by follow-up duration and intervention type, no subgroup analysis was conducted based on different depression assessment scales. Additionally, data from multiple time points of the same trial were included as independent entries and counted toward the total sample size, which may have introduced bias in the overall estimates [8].

SCI not only causes profound neurological impairments but also gives rise to multiple complications that substantially affect patients’ functional recovery, mental health, quality of life, and sleep. With an increasing number of RCTs investigating the potential benefits of telemedicine in SCI, it is crucial to clarify the extent to which patients may benefit from such interventions, thereby promoting their broader application in clinical practice. Therefore, the objective of this study was to systematically review and meta-analyze the effects of telemedicine interventions on psychological health, health-related quality of life, sleep-related functioning, functional independence and rehabilitation outcomes, and pain intensity in patients with SCI, aiming to provide robust evidence for clinical decision-making and to guide the optimization of tele-rehabilitation strategies in this population.

## Methods

### Protocol and Registration

The protocol for this systematic review was registered with the International Prospective Register of Systematic Reviews (PROSPERO; registration number: CRD420251149977).

### Eligibility Criteria

Eligibility criteria were established based on the PICOS (Population, Intervention, Comparison, Outcome, and Study Design) framework. The study population included patients diagnosed with SCI, with no restrictions on sex, age, injury level, or severity. Eligible interventions were defined as telemedicine-related modalities, including video consultations, mobile health apps, remote monitoring, and telephone follow-up. Comparators were not restricted and could include usual care or no tele-intervention. Outcomes were prespecified in five domains, including psychological health (eg, depression and/or anxiety), health-related quality of life, sleep-related functioning, functional independence, and rehabilitation outcomes, and pain intensity. Trials contributed to meta-analysis only when outcome data for at least one prespecified domain were available and extractable; otherwise, they were included in the descriptive synthesis. Only RCTs were eligible. To ensure that comparisons reflected allocation to mode of care delivery rather than self-selection, we included only trials in which participants were randomized to receive a telemedicine-delivered intervention versus a comparator condition (eg, usual care, standard follow-up, or in-person care). Studies in which telemedicine exposure was determined primarily by participant preference, acceptance of an offer, or other nonrandom allocation mechanisms were excluded. In line with the search strategy, studies were restricted to English-language full-text reports.

The exclusion criteria were as follows: (1) nonprimary research articles, including systematic reviews, narrative commentaries, conference abstracts, and case reports, and other non-RCT designs; (2) interventions not related to telemedicine; and (3) duplicate publications or studies with overlapping datasets. These exclusions were applied to ensure methodological rigor and extractable data.

Meta-analysis was performed when quantitative data were extractable; otherwise, findings were synthesized narratively. Trials not reporting any prespecified outcome domain were included in the descriptive summary (Table 1) but were not included in the quantitative synthesis. When outcomes were measured but insufficiently reported for extraction, authors were contacted where feasible, and missing results were documented.

**Table 1.** Baseline features and design elements of included studies.

Study	Country	Study design	Sample size	Sex (male), n (%)	Age (year)	Interventions	Duration	Outcomes	Follow-up
Arora et al (2017) [9] / Arora et al (2017) [10] (same RCT^a^; 2 reports)	India and Bangladesh	RCT^a^	INT^b^: 60; CON^c^: 60	INT:52 (87); CON: 54 (90)	INT: mean 35 (SD 11); CON: mean 36 (SD 12)	INT: weekly phone calls from a trained health-care professional; CON: usual care	12 weeks	Size of the pressure ulcer; PUSH^d^; Depth of PU^e^; Undermining distance of PU; Braden scale; HADS^f^; WHODAS^g^; EQ-5D-5L^h^; Self-rated health EQ-5D-VAS^i^; Participants’ impression of PU status; Participants’ confidence to manage PU; Clinician’s impression of PU status; Participants’ satisfaction; Self-report time for PU resolution; Reduction in the size of pressure ulcer; EQ-5D-5L; Cost of delivering the intervention; Productivity cost	12 weeks
Badr et al (2024) [11]	United States	RCT	INT: 32; CON: 31	INT: 29 (91); CON: 29 (94)	INT: mean 61.6 (SD 10.0); CON: mean 59.8 (SD 10.4)	INT: education about sleep-disordered breathing (SDB) with positive airway pressure (PAP), goal setting, troubleshooting, and motivational enhancement; CON: nondirective sleep education only	3 months	PAP use; PSQI^j^; CHART^k^; WHOQOL^l^; PHQ-9^m^; FFS^n^; ESS	1, 3, and 6 months
Bombardier et al (2021) [12]	United States	Pilot RCT	INT: 7; CON: 8	INT: 5 (71); CON: 6 (75)	INT: mean 56 (SD 13); CON: mean 49 (SD 13)	INT: 16-session Diabetes Prevention Program delivered via telephone; CON: usual care	24 weeks	VO_2 peak_^o^; BMI; DXA; ISI^p^; PARA-SCI^q^; Brief Pain Inventory intensity and interference scales; Wheelchair User’s Shoulder Pain Index; PHQ-9; SF-12^r^; WHOQOL- BREF^s^; Exercise self-efficacy	6 months
Bombardier et al (2023) [13]	United States	RCT	INT: 89; CON: 85	INT: 63 (71); CON: 70 (82)	INT: mean 47.8 (SD 15); CON: mean 47.5 (SD 14.8)	INT: assessment, medical care coordination, adherence support, outcome monitoring, and decision support, along with brief psychological interventions to the patients via up to 12 in-person or telephone sessions; CON: usual care	4 months	WHOQOL-BREF; NRS^t^; PHQ-9, HSCL-20B; LTPAQ-SCI^u^	4 and 8 months
Burke et al (2019) [14]	Ireland	RCT	INT: 35; CON: 34	INT: 25 (71%); CON: 27 (79%)	INT: mean 50 (SD 12.3); CON: mean 52 (SD 13.8)	INT: 6 module cognitive behavioral therapy pain management programs (CBT‐PMPs) weekly; CON: usual care	6 weeks	WHOQOL‐BREF; ISCIPBDS^v^; DN4^w^; CPAQ‐8^x^; BPI^y^; HADS; PSQI	6 weeks and 3 months
Carlson et al (2017) [15]	United States	RCT	INT: 83; CON: 87	INT: 72 (84.1); CON: 69 (85.7)	INT: mean 41.7 (SD 12.9); CON: mean 42.5 (SD 12.2)	INT: 12-month lifestyle-based treatment administered by health care professionals, largely via in-home visits and phone contacts; CON: usual care	12 months	MSPrIs^z^; SF-36^aa^; SWLS^ab^; Quasi-Adaptive Short Form for the Patient Reported Outcomes Measurement Information System (PROMIS) Version 1 Depression Item Bank; ISEL^ac^; Pressure Ulcer Knowledge Test; Adapted Moorong Self Efficacy Scale; Adapted Cut Down, Annoyed, Guilty, and Eye Opener (CAGE) Questionnaire	12 and 24 months
Chantanachai et al (2025) [16]	Thailand	RCT	INT^b^: 15; CON^c^: 15	INT: 10 (66.7) CON: 9 (60)	INT: mean 42.7 (SD 11.6); CON: mean 41.2 (SD 12.8)	INT: 20 min of active-tDCS, followed by a 1 h exercise program; CON: 20 min of sham-tDCS, followed by a 1 h exercise program, 12 sessions (3 times a week)	4 weeks	ISNCSCI^ad^; SCIM^ae^; TAI^af^; WHOQOL-BREF–Thai; H-reflex; m-MAS^ag^; Upper limb muscle strength test	1 month
Chemtob et al (2019) [17]	Canada	Pilot RCT	INT: 10; CON: 12	All: 16 (72.7)	All: mean 51.64 (SD 12.13)	INT: 1 h counseling session per week via an online video-chat platform; CON: regular routine	8 weeks	SDT^ah^; LTPA^ai^; MVPA^aj^; Life Satisfaction Questionnaire-11; PHQ-9; Meaning Questionnaire	6 and 10 weeks
Coulter et al (2017) [18]	United Kingdom	Pilot RCT	INT: 15; CON: 8	INT: 9 (56); CON: 5 (63)	INT: mean 51.5 (SD 13.0); CON: mean 48.1 (SD 10.6)	INT: web-based physiotherapy twice per week; CON: usual care	8 weeks	6MPT^ak^, 6MWT^al^; work HR–resting HR; Borg RPE; HADS; WHO-QOL BREF	8 weeks
Dallolio et al (2008) [19]	Belgium, Italy, and United Kingdom	RCT	INT: 69; CON: 68	All: 107 (84.25)	All: median 40 (IQR 18-85)	INT: telemedicine weekly sessions in the first 2 months, followed by biweekly telemedicine sessions for 4 months; CON: standard care	6 months	FIM^am^; SCIM; clinical outcomes; patient satisfaction questionnaire	2 and 6 months
Dorstyn et al (2012) [20]	Australia	RCT	INT: 20; CON: 19	INT: 13 (65); CON: 14 (74)	INT: mean 53.8 (SD 16.3); CON: mean 53.1 (SD 20.0)	INT: Seven telecounseling sessions were delivered over 12 weeks; CON: standard care	12 weeks	DASS21^an^; M.I.N.I.^ao^; SCL CSQ^ap^; SCL EWB^aq^; MDSS^ar^; Cost-effectiveness	12 weeks and 3 months
Dorstyn et al (2019) [21]	Australia	Pilot RCT	INT: 25; CON: 23	INT: 12 (48); CON: 15 (65.2)	INT: mean 43.0 (SD 10.9); CON: mean 40.7 (SD 11.0)	INT: provided job-searching and career planning information through text, videos, and interactive activities; CON: wait-list control	4 weeks	JSES^as^; LOT-R^at^; PHQ-9	4 weeks
Hearn et al (2018) [22]	United Kingdom	RCT	INT: 36; CON: 31	INT: 17 (47); CON: 14 (45)	INT: mean 43.8 (SD 8.7); CON: mean 45.2 (SD 12.2)	INT: online mindfulness intervention; CON: internet-delivered psychoeducation	8 weeks	HADS; WHOQoL-BREF; FFMQ25^au^; NRS; PCS^av^, Retention rates	8 weeks and 3 months
Hossain et al (2016) [23]	Bangladesh	Pilot RCT	INT: 15; CON: 15	INT: 13 (86.7); CON: 13 (86.7)	INT: median 29 (IQR 24-35); CON: median 34 (IQR 23-36)	INT: regular telephone contact and 3 home visits over 2 years; CON: usual care	2 years	all-cause mortality; SCI-SCS^aw^; Presence of pressure ulcers; CES Depression Scale^ax^; SF12; PUSH; SCIM; WHODAS	2 years
Hossain et al (2021) [24]	Bangladesh	RCT	INT: 204; CON: 206	INT: 181 (89); CON: 188 (91)	INT: median 31.4 (IQR 24.5‐41.0); CON: median 33.4 (IQR 25.7‐45.0)	INT: 36 phone calls and 3 home visits over the first 2 years following discharge; CON: usual care	2 years	Death; SCI-SCS; PUSH; CESD-R^ay^; WHODAS; SF12; SCIM	2 years
Houlihan et al (2017) [25]	USA	RCT	INT: 42; CON: 42	INT: 30 (71.4); CON: 32 (76.2)	INT: mean 44.0 (SD 13.4); CON: mean 47.5 (SD 14.4)	INT: 42 experimental subjects on a tapered call schedule; CON: usual care	6 months	PAM^az^; Social and Role Activities Limitations; Quality of life; Communication With Physicians Scale; Patient Satisfaction Scale; Global rating of change	2, 4, and 6 months
Irgens et al (2022) [26]/ Irgens et al 2024 [27] (same RCT; reports)	Norway	RCT	INT: 28; CON: 28	INT: 24 (86); CON: 21 (78)	INT: mean 58 (SD 14); CON: mean 58 (SD 13)	INT: additional videoconference (VCG); CON: regular care	1 year	QALYs^ba^; HRQoL^bb^; Cost measurement; environmental analysis; Michelin Travel’s Route Planner; PI healing^bc^, Interaction, Satisfaction, and Safety	1 year
Kowalczewski et al (2011) [28]	Canada	RCT (crossover design)	INT: 7; CON: 6	All: 7 (54)	All: mean 35.92 (SD 11.96)	INT: “ReJoyce ET” comprised FES-ET on a workstation, tele-supervised 1 h/d, 5 d/week; CON: conventional exercise therapy, tele-supervised 1 h/d, 5 d/week	6 weeks	ARAT^bd^; grasp and pinch forces; RAHFT^be^	6, 16, and 30 weeks
Kryger et al (2019) [29]	United States	RCT	INT: 19; CON: 19	INT: 13 (68); CON: 12 (63)	INT: mean 37.9 (SD 13.4); CON: mean 44.1 (SD 15.3)	INT: 30 min of training to use the Interactive Mobile Health and Rehabilitation (iMHere) app; CON: standard care	9 months	UTIs^bf^; Number of pressure injuries; ED visits^bg^; Number of hospitalizations; COPM^bh^; Adolescent Self-Management and Independence Scale; BDI-II^bi^; Patient Assessment of Chronic Illness Care; WHOQOL; Craig Handicap Assessment and Reporting Technique Short Form	3, 6, and 9 months
Lawrason et al (2023) [30]	Canada	RCT	INT: 12; CON: 9	INT: 7 (58.3); CON: 5 (55.6)	INT: mean 37.15 (SD 10.12); CON: mean 43.37 (SD 12.67)	INT: SCI Step Together, features weekly programming; CON: continue normal daily activity	8 weeks	LTPA; Quality participation	4 and 8 weeks
Li et al (2021) [31]	China	RCT	INT: 39; CON: 39	All: 40 (51.3)	All: mean 47.12 (SD 10.90)	INT: continuous care via the WeChat platform; CON: regular care	6 months	FAD^bj^; HPLPII^bk^	3 and 6 months
Li et al (2021) [32]	China	RCT	INT: 40; CON: 40	INT:28 (70); CON: 26 (65)	INT: median 57.8 (IQR 23‐72); CON: median 59.1 (IQR 17‐69)	INT: online home nursing care; CON: routine discharge guidance	12 months	Complications; ODI^bl^; SF-36	6 and 12 months
Liu et al (2021) [33]	China	RCT	INT: 49; CON: 49	INT: 41 (84); CON: 40 (82)	INT: mean 40.37 (SD 12.18); CON: mean 43.06 (SD 12.06)	INT: 5 follow-ups conducted by trained nurses through the app, which had 4 core functions; CON: a routine telephone follow-up	12 weeks	MSES^bm^; SF-36	12 and 24 weeks
Liu et al (2023) [34]	China	RCT	INT: 49; CON: 49	INT: 41 (83.7); CON: 40 (81.6)	INT: mean 40.37 (SD 12.18); CON: mean 43.06 (SD 12.06)	INT: 5 sessions nurse-led multidisciplinary team via APP; CON: one routine telephone counseling	12 weeks	BDI-II	12 and 24 weeks
Mackelprang et al (2016) [35]	United States	RCT	INT: 85; CON: 83	INT: 70 (82); CON: 63 (76)	INT: mean 40.4 (SD 15.7); CON: mean 42.0 (SD 16.0)	INT: eleven 30-45-minute scheduled telephone calls to provide education, resources, and support; CON: usual care	10 months	health care use and medical complications composite; PHQ-9; Delighted-Terrible Scale; EuroQol thermometer; Subjective health using a single item from the SF-36; CHART-SF^bn^	3, 6, 9, and 12 months
Mercier et al (2015) [36]	United States	Pilot RCT	INT: 53; CON: 53	INT: 42 (79.2); CON: 34 (64.2)	INT: mean 45.8 (SD 12.1); CON: mean 45.0 (SD 14.0)	INT: CareCall intervention of regular automated phone calls that delivered educational content and peer and clinical expert perspectives; CON: usual care and the resource book	6 months	PHQ-9; PUSH; Health care use; CHART-SF; Engaging with the CareCall system	6 months
Migliorini et al (2016) [37]	Australia	RCT	INT: 34; CON: 25	INT: 25 (74); CON: 17 (68)	INT: mean 47.5 (SD 12.2); CON: mean 52.8 (SD 12.9)	INT: Electronic Personal Administration of Cognitive Therapy (ePACT); CON: waitlist control telephone interviews	10‐12 weeks	DASS21; Personal Well-being Index–Adult; SCL EWL^bo^;	3 and 6 months
Pain et al (2007) [38]	UK, Italy, and Belgium	RCT	INT: 38; CON: 39	All: 64 (82.8)	All: median 42 (IQR 19-86)	INT: Internet-based video-link technology; CON: standard support	6 months	WHO QoL-100, HADS	2 and 6 months
Rintala et al (2008) [39]	United States	RCT	INT 1: 20; CON 1: 11; CON 2: 10	INT: 20 (100); CON 1: 11 (100); CON 2: 10 (100)	INT 1: mean 54.82 (SD 10.85); CON 1: mean 50.64 (SD 8.83); CON 2: mean 53.86 (SD 13.63)	INT 1: pressure ulcer education and monthly structured telephone follow-up; CON 1: monthly mail or telephone follow-up without educational content; CON 2: quarterly mail or telephone follow-up without educational content	24 months	Time to pressure ulcer recurrence;	24 months
Swarnakar et al (2023) [40]	India	RCT	INT: 15; CON: 15	INT: 12 (80); CON: 14 (93.3)	INT: mean 28.2 (SD 6.9); CON: mean 26.3 (SD 7.7)	INT: biweekly telerehabilitation sessions; CON: usual care	8 weeks	SCIM; CAS^bp^	4 and 8 weeks
Worobey et al (2018) [41]	United States	RCT	INT 1: 10; INT 2: 39; CON: 22	INT 1: 6 (60); INT 2: 31 (79.5); CON: 21 (95.5)	INT 1: median 53.5 (IQR 47.0‐55.0); INT 2: median 54.0 (IQR 45.0‐61.0) CON: median 56.0 (IQR 54.0‐58.0)	INT 1: Individualized, in-person transfer training session; INT 2: a web-based transfer training module; CON: web training at their follow-up visit	6 months	TAI	2, 6, and 12 months
Jaglal et al (2026) [42]	Canada	Pilot RCT	INT: 31; CON: 34	INT: 17 (57); CON: 13 (43)	INT: mean 49.6 (SD 11.1); CON: mean 48.7 (SD 14.1)	INT: web-based peer health-coaching self-management program: up to 14×1 h online video coaching sessions, goal setting, and action planning, and sortable resource library; CON: usual care	6 months	self-management skills (Health Education Impact Questionnaire et al); rehospitalization days; secondary conditions, self-efficacy; HRQoL; social and role limitations etc	6 and 12 months
Young et al (2025) [43]	United States	RCT	INT 1: 12; INT 2: 12; CON (AC): 12	INT 1: 6 (50.0); INT 2: 7 (58.3); CON: 4 (33.3)	INT 1: mean 42.0 (SD 11.6); INT 2: mean 47.7 (SD 11.8); CON: mean 46.4 (SD 12.0)	INT 1: movement-to-music teleexercise via prerecorded videos 3×/week; INT 2: standard exercise training prerecorded videos 3×/week; CON: weekly educational articles (attention control)	8 weeks	feasibility (recruitment and retention), usability (^bq^SUS, ^br^Health-ITUES + interviews), acceptability (^bs^PACES-8 + interviews); preliminary outcomes: ^bt^LTPAQ-SCI, ^bu^PROMIS short forms	8, 12, and 16 weeks

a RCT: randomized clinical trial.

b INT: intervention.

c CON: control.

d PUSH: Pressure Ulcer Scale for Healing.

e PU: pressure ulcer.

f HADS: Hospital Anxiety and Depression Scale.

g WHODAS: World Health Organization Disability Assessment Schedule.

h EQ-5D-5L: Euro Quality of Life 5-dimensional 5-level.

i VAS: Visual Analog Scale.

j PSQI: Pittsburgh Sleep Quality Index.

k CHART: Craig Handicap Assessment and Reporting Technique.

l WHOQOL: World Health Organization Quality-of-Life Scale

m PHQ-9: Patient Health Questionnaire-9.

n FFS: Flinders Fatigue Scale.

o VO_2 peak_: peak oxygen consumption.

p ISI: Insulin sensitivity index.

q PARA-SCI: Physical Activity Recall Assessment for SCI.

r SF-12: Medical Outcomes Study Short Form-12.

s WHOQOL-BREF: World Health Organization Quality of Life-BREF Scale.

t NRS: numerical rating scales.

u LTPAQ-SCI: Leisure Time Physical Activity Questionnaire for SCI.

v ISCIPBDS: International Spinal Cord Injury Pain Basic Data Set.

w DN4: Douleur Neuropathique en 4 Questions.

x CPAQ‐8: Chronic Pain Acceptance Questionnaire‐8.

y BPI: Brief Pain Inventory.

z MSPrIs: Medically serious pressure injuries.

aa SF-36: 36-item short-form health survey.

ab SWLS: Satisfaction with Life Scale.

ac ISEL: Interpersonal Support Evaluation List.

ad ISNCSCI: Neurological Classification of Spinal Cord Injury.

ae SCIM: spinal cord independence measure.

af TAI: transfer assessment instrument version 4.0.

ag m-MAS: modified-Modified Ashworth Score.

ah SDT: self-determination theory.

ai LTPA: leisure-time physical activity.

aj MVPA: moderate to vigorous LTPA.

ak 6MPT: 6-minute Push Test.

al 6MWT: 6-minute Walk Test.

am FIM: Functional Independence Measure.

an DASS-21: Depression Anxiety Stress Scale-21.

ao MINI: Mini International Neuropsychiatric Interview.

ap SCL CSQ: Spinal Cord Lesion Coping Strategies Questionnaires.

aq SCL EWB: Spinal Cord Lesion Emotional Well-Being Questionnaire.

ar MDSS: Multidimensional Measure of Social Support.

as JSES: Job Procurement Self-Efficacy Scale.

at LOT-R: Life Orientation Test-Revised.

au FFMQ25: Five Facet Mindfulness Questionnaire.

av PCS: The Pain Catastrophizing Scale.

aw SCI-SCS: Spinal Cord Injury Secondary Conditions Scale.

ax CES Depression Scale: Center for Epidemiologic Studies Depression Scale.

ay CESD-R: Center for Epidemiologic Studies Depression Scale revised version.

az PAM: Patient Activation Measure.

ba QALYs: quality-adjusted life years.

bb HRQoL: health-related quality of life.

bc PI healing: Pressure Injuries healing.

bd ARAT: Action Research Arm Test.

be RAHFT: ReJoyce automated hand function test.

bf UTIs: urinary tract infections.

bg ED visit: emergency department visit.

bh COPM: Canadian Occupational Performance Measure.

bi BDI-II: Beck Depression Inventory-II.

bj FAD: Family Assessment Device.

bk HPLPII: Health Promoting Lifestyle Profile.

bl ODI: Oswestry Disability Index.

bm MSES: Moorong Self-Efficacy Scale.

bn CHART-SF: Craig Handicap Assessment and Reporting Technique Short Form.

bo SCL EWL: Spinal Cord Lesion Emotional Well-being Questionnaire version 1 Australia.

bp CAS: Coronavirus Anxiety Scale.

bq SUS: System Usability Scale.

br Health-ITUES: Health Information Technology Usability Evaluation Scale.

bs PACES-8: Physical Activity Enjoyment Scale–8 item version.

bt LTPAQ-SCI: Leisure Time Physical Activity Questionnaire for People with Spinal Cord Injury.

bu PROMIS: Patient-Reported Outcomes Measurement Information System.

bv ESS: Epworth Sleepiness Scale.

### Information Sources

To ensure comprehensive identification of relevant studies, we searched PubMed (via the NLM interface), Web of Science Core Collection (via Clarivate), Embase (via Ovid), MEDLINE (via Ovid), and the Cochrane Central Register of Controlled Trials (CENTRAL; via the Cochrane Library). The final search update was performed on February 17, 2026. In addition to database searching, we also searched trial registries and conducted supplementary searches (citation searching, websites and search engines, and hand searching). We additionally performed backward and forward citation searching using the reference lists of included studies and relevant reviews, and the “cited reference” function in Web of Science. Searches were restricted to English-language full-text reports. The search methods and reporting followed the PRISMA-S (Preferred Reporting Items for Systematic Reviews and Meta-Analyses–Search) extension [44].

### Search Strategy

A comprehensive strategy was developed by combining controlled vocabulary (eg, MeSH and Emtree terms) with free-text terms and adapting syntax to each database. Search concepts included (1) spinal cord injury and related terms, (2) telemedicine-related modalities (including telerehabilitation, telerehab, and closely related telehealth and telemedicine terms), and (3) an RCT component. To avoid overly broad retrieval, broad digital-delivery terms, such as online, telephone, app-based, web-based, and internet-based terms, were combined with rehabilitation- or therapy-related terms. An RCT filter was applied where appropriate, and animal-only records were excluded. The full, reproducible search strategies for all databases, copied exactly as run (including filters and limits), are provided in Table S1 in Multimedia Appendix 1. No natural language processing or text-mining tools were used to generate or refine search terms. No automated search translation tools were used; search strings were adapted manually to each database. The PRISMA (Preferred Reporting Items for Systematic Reviews and Meta-Analyses) flow diagram has been updated accordingly (Checklist 1). All records were imported into EndNote for record management and deduplication before screening. The electronic search strategy underwent internal checking for syntax and completeness. When outcome data were measured but incompletely reported, study authors were contacted when feasible and necessary to obtain missing information.

### Selection Process

Two reviewers (WZ and LH) conducted the study selection process in a 2-phase approach, initial screening of titles and abstracts to assess relevance, followed by full-text evaluation to determine final eligibility. Reference management software (EndNote) was used to eliminate duplicate records, and all screening decisions and reasons for exclusion were systematically documented. Any discrepancies between reviewers were resolved through consensus discussions involving a third reviewer. No additional information was sought from investigators, and no automation tools were used in the selection process. No translation was required because searches were restricted to English-language full-text reports.

### Data Collection Process

The data extraction process was independently conducted by 2 reviewers (WZ and LH) using a predesigned Microsoft Excel spreadsheet. Extracted information included basic study details (author, year of publication, and country), study design, sample size, participant characteristics (eg, sex and age), type of intervention and control group settings, outcomes, and follow-up duration. Any discrepancies in extracted data were resolved through discussion, with a third reviewer adjudicating when necessary. In cases of missing or unclear data, attempts were made to contact the original authors for clarification. All extracted data were cross-verified to ensure accuracy and consistency. No automation tools were used for data extraction, and no translation was required because inclusion was restricted to English-language full-text reports. No software was used to extract data from figures. When multiple reports corresponded to the same study, data were collated and extracted as a single study; where overlapping or inconsistent information was identified, the most complete report and/or the longest follow-up was prioritized.

### Data Items

We focused on five core outcome domains:

1. Psychological health, including depression and anxiety, was assessed using validated instruments such as the Patient Health Questionnaire-9 (PHQ-9), Beck Depression Inventory (BDI), Center for Epidemiologic Studies Depression Scale (CES-D), and Hospital Anxiety and Depression Scale (HADS).
2. Health-related quality of life, measured using the World Health Organization Quality of Life-BREF (WHOQOL-BREF), EuroQol Five Dimensions Questionnaire (EQ-5D), and Short Form Health Survey-36 (SF-36).
3. Sleep-related functioning was evaluated using the Pittsburgh Sleep Quality Index (PSQI) and Epworth Sleepiness Scale (ESS).
4. Functional independence and rehabilitation outcomes were assessed using the Spinal Cord Independence Measure (SCIM), Functional Independence Measure (FIM), and Craig Handicap Assessment and Reporting Technique (CHART).
5. Pain intensity, measured using the Numerical Rating Scale (NRS).

For each outcome domain, we extracted all relevant measurement instruments, assessment time points, and available analytical results. For meta-analysis, we prioritized postintervention values. For meta-analysis, we prioritized postintervention values at the end of the intervention; when multiple postintervention time points were available, we prioritized the end-of-intervention time point and summarized other time points narratively and explored them in sensitivity analyses when appropriate. Effect estimates were calculated respecting each instrument’s original scale without recoding. No changes were made to outcome domain definitions or selection rules after protocol registration. When required information was missing or unclear and could not be confirmed with the study authors, it was recorded as not reported, and no unverifiable assumptions were made.

### Study Risk of Bias Assessment

The risk of bias was evaluated using the Cochrane Risk of Bias 2 (version 2; RoB 2; 22 August 2019 guidance), implemented using the RoB 2 Excel tool (ROB2_IRPG beta v9), a structured framework designed to assess the internal validity of trial outcome results. RoB 2 addresses five distinct domains: (1) bias related to the randomization procedure; (2) bias resulting from deviations from the intended intervention; (3) bias associated with incomplete outcome data; (4) bias in the assessment of outcomes; and (5) bias stemming from selective reporting of results. Each domain is appraised through a standardized decision algorithm, culminating in an overall judgment for each outcome, classified as “low risk,” “some concerns,” or “high risk.” Two reviewers independently assessed risk of bias for all included trials, with disagreements resolved by discussion and third-reviewer adjudication when needed. No adaptations were made to the RoB 2 tool, no additional information was sought from study investigators for risk-of-bias assessment, and no automation tools were used. Risk-of-bias judgments informed sensitivity analyses and the certainty of evidence appraisal.

### Data Analysis

#### Effect Measures

All outcomes synthesized quantitatively were continuous. We used mean difference (MD) when outcomes were measured using the same instrument and standardized mean difference (SMD) when different instruments were used within the same outcome domain. Results were reported with corresponding 95% CIs, which were used to quantify the uncertainty around the average pooled effect. No prespecified thresholds were used to categorize effect sizes, and synthesized results were not re-expressed into alternative effect measures.

#### Synthesis Methods

Studies were grouped for synthesis by the 5 predefined outcome domains. Within each domain, studies were considered eligible for meta-analysis when interventions and outcome constructs were sufficiently comparable and postintervention data were extractable; otherwise, findings were synthesized narratively. We addressed unit-of-analysis issues by ensuring that each participant sample contributed only once to any given meta-analysis and by linking multiple reports of the same trial to a single study, thereby avoiding double-counting and correlated data [45]. Data were prepared for synthesis by prioritizing end-of-intervention postintervention values when multiple follow-up time points were reported and by harmonizing effect direction across instruments; when required, summary statistics were missing, authors were contacted where feasible and otherwise studies were not pooled for that outcome. Given anticipated clinical and methodological heterogeneity across trials, all meta-analyses used a random-effects model [46]. Random-effects inference was based on the Hartung-Knapp-Sidik-Jonkman method, and between-study variance was estimated using restricted maximum likelihood [47]. Heterogeneity was quantified using Cochran Q and the between-study variance (τ²) and its square root (τ); *I*² was reported as a descriptive statistic and considered in interpretation, but not used to choose the meta-analysis model. Confidence intervals were used to quantify uncertainty around the average pooled effect. To aid interpretation of how true effects might vary across settings, 95% prediction intervals were additionally reported for main meta-analyses, but not for subgroup meta-analyses. Prediction intervals were reported when at least 5 studies were available; for analyses including 5‐9 studies, they were interpreted cautiously, whereas for analyses with fewer than 5 studies they were not reported [48]. We additionally conducted exploratory subgroup analyses for depressive symptoms, stratified by intervention duration, depression measurement instrument, and follow-up time point, to explore potential sources of between-study heterogeneity. Analyses were implemented in R (*meta package*; R Foundation for Statistical Computing).

#### Reporting Bias Assessment

Potential reporting biases and small-study effects were assessed using funnel plot asymmetry when at least 10 studies were available for an outcome meta-analysis. When fewer than 10 studies were available, formal assessment of funnel plot asymmetry was not performed due to limited power and this limitation was noted. Two reviewers (WZ and RL) assessed funnel plot asymmetry independently, with a third reviewer adjudicating when needed. The Egger test was not performed. No additional information was sought from study investigators specifically for this assessment, no automation tools were used, and no dedicated tool for bias due to missing results was applied. Funnel plot asymmetry was interpreted as evidence of small-study effects, for which publication bias is only one of several possible explanations [49].

#### Certainty Assessment

The certainty of evidence for each key outcome was assessed using the GRADE approach (Grading of Recommendations Assessment, Development, and Evaluation). Two reviewers (WZ and LW) independently rated the certainty of evidence as high, moderate, low, or very low, considering risk of bias, inconsistency, indirectness, imprecision, and publication bias. Disagreements were resolved through discussion with third-reviewer adjudication. GRADE judgment and certainty ratings were reported alongside pooled results in the Results section.

### Ethical Considerations

Ethical approval is not applicable. This study is a systematic review and meta-analysis of previously published studies and did not involve the collection of individual-level data.

## Results

### Study Selection

**Figure 1.**
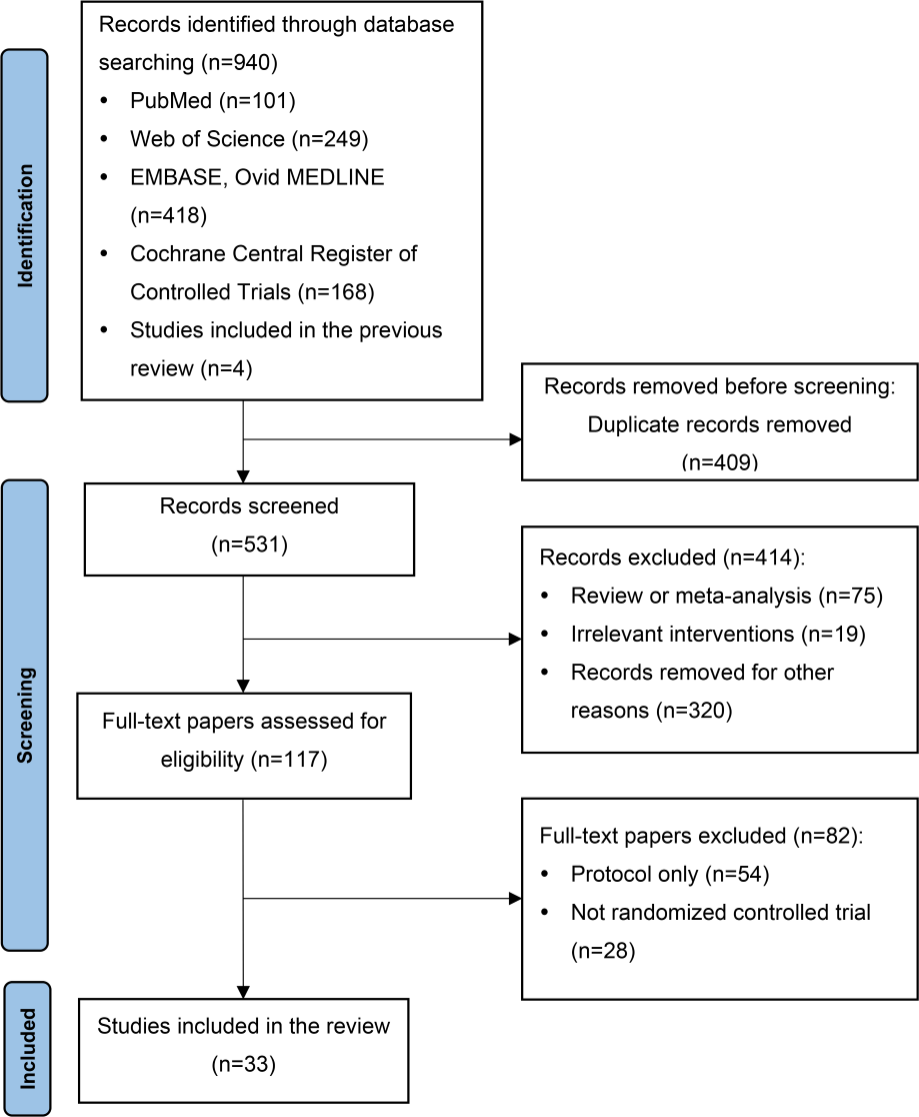
PRISMA (Preferred Reporting Items for Systematic Reviews and Meta-Analyses) flowchart.

A total of 940 records were identified through comprehensive database searches, including PubMed (n=101), Web of Science (n=249), EMBASE and Ovid MEDLINE (n=418), and the Cochrane Central Register of Controlled Trials (n=168), with an additional 4 records identified from prior systematic reviews (n=4). After deduplication using reference management software, 531 unique records remained for title and abstract screening, of which 414 were excluded (reviews or meta-analyses, n=75; irrelevant interventions, n=19; other records not meeting the predefined inclusion criteria, n=320). A total of 117 reports were sought for full-text retrieval, and all were retrieved (n=0). After full-text assessment, 82 reports were excluded due to study protocols without results (n=54) or lack of an RCT design (n=28). Ultimately, we included 33 studies (35 reports) [9-43]. Two studies had multiple reports (Arora et al 2017 [9]/2017 [10]; Irgens et al 2022 [26]/2024 [27]). The study selection process is shown in Figure 1.

### Study Characteristics

We included 33 studies (35 reports) [9-43]; 2 studies had multiple reports (Arora et al 2017/2017 [9,10] clinical and economic evaluations; Irgens et al 2022/2024 [26,27] clinical and cost-utility and environmental analyses), while all other studies were single reports. Sample sizes ranged from 13 participants to >400, with participants predominantly male, and reported mean or median ages generally spanning adulthood to older age. Intervention duration varied from single-session training to 8-12 weeks, to 3-6 months, and up to 2 years in community follow-up programs. Follow-up commonly occurred at the end of treatment and at short-term time points such as 1-3 months. Some studies extended follow-up to 6-12 months or longer. Full study characteristics are presented in Table 1.

The included studies used a diverse array of remote or hybrid intervention modalities. Telephone-based interventions were the most frequently used modality, accounting for 27.3% of studies (9/33) [9,11-13,20,35,36,39]. These interventions typically involved scheduled calls by health care professionals to deliver health consultations, psychological counseling, or disease-specific education. In some cases, telephone contact was supplemented with home visits to enhance personalization and continuity of care (9.1%, 3/33) [15,23,24]. Web-based or mobile app and social-platform interventions accounted for 18.2% of the included studies (6/33) [29-34]. These platforms, such as WeChat (Tencent), mobile apps, and dedicated websites, facilitated ongoing care, rehabilitation guidance, interactive education, and multidisciplinary support. These interventions were typically led by nurses or rehabilitation teams. Videoconferencing-based interventions were used in 15.2% of studies (5/33) [17,19,26,27,38,42], offering real-time interaction and visual feedback for psychological support, rehabilitation, and educational guidance. Tele-supervised rehabilitation or teleexercise programs also accounted for 15.2% of studies (5/33) [16,18,28,40,43] and were delivered through remote supervision and periodic reassessment, sometimes combined with adjunct technologies. A minority of studies investigated novel intervention modalities, such as transcranial direct current stimulation (tDCS) and functional electrical stimulation exercise training (FES-ET), reflecting the evolving landscape of remote therapeutic strategies (eg, Chantanachai et al [16] and Kowalczewski et al [28]). Psychological and behavioral interventions were widely represented, including cognitive behavioral therapy (CBT), mindfulness-based training, vocational planning, and electronic cognitive therapy (eg, Burke et al [14]; Hearn et al [22]; and Migliorini et al [37]). Full details of intervention modalities for each study are provided in Table 1.

### Risk of Bias Assessment

The risk of bias across included studies was assessed using the Cochrane RoB 2 tool, and the results are summarized in Figure S1 in Multimedia Appendix 1. Multimedia Appendix 1 presents study-level judgments across the 5 RoB 2 domains (randomization process, deviations from intended interventions, missing outcome data, measurement of the outcome, and selection of the reported result) together with an overall judgment. Overall, a substantial proportion of included studies were rated as having some concerns, and a smaller subset was judged to be at high risk of bias, while approximately half were rated as low risk overall. Figure S1B Multimedia Appendix 1 shows the distribution of risk of bias judgments by domain. Missing outcome data had the highest proportion of low-risk judgments (approximately 94%), indicating that incomplete outcome data were generally well addressed in most trials. The randomization process domain also showed a high proportion of low-risk judgments (approximately 71%), suggesting that sequence generation and allocation procedures were relatively robust in many studies. In contrast, deviations from intended interventions and selection of the reported result were more frequently rated as some concerns, reflecting limitations related to intervention adherence, analytic approach, or reporting transparency. Measurement of the outcome showed a more mixed pattern, with a notable proportion of studies judged to be at high risk (approximately 23%), consistent with challenges related to blinding, outcome ascertainment, or reliance on self-reported measures. Taken together, these results indicate that concerns were driven primarily by issues related to deviations, outcome measurement, and selective reporting rather than missing data.

### Results of Individual Studies and Syntheses

#### Psychological Health

**Figure 2.**
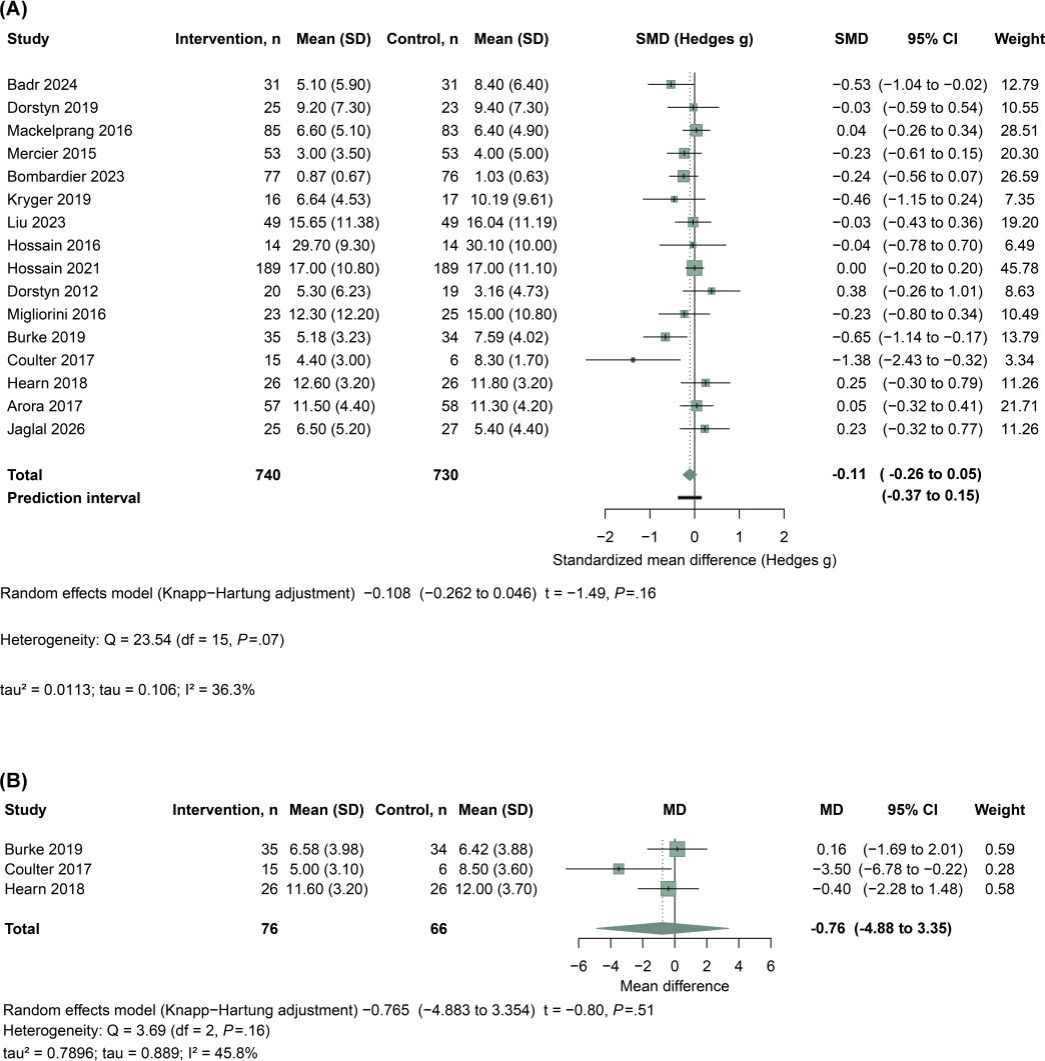
Forest plots show (A) depression and (B) anxiety. Each study reports n, mean, and SD for intervention and control groups and the study effect with 95% CI. Depression is summarized as standardized mean difference (SMD). Hedges *g* and anxiety as standardized mean difference (MD). Random-effects models were used. Heterogeneity is reported as Q, tau, tau², and *I*². Negative values favor the intervention [9,11,13,14,18,20-24,29,34-37,42].

This systematic review synthesized evidence from 16 studies [9,11,13,14,18,20-24,29,34-37,42] evaluating the efficacy of telehealth interventions in mitigating depressive symptoms among individuals with SCI. For each study, Figure 2A presents group-level summary statistics for the intervention and control groups, including sample size, mean, and SD, together with the study-specific effect estimate and its 95% CI. Due to heterogeneity in depression assessment instruments across studies, SMD was used as the effect size metric, and pooled estimates were calculated using a random-effects model. Individual study effects varied in direction and magnitude, with several trials suggesting reductions in depressive symptoms and others showing little or no difference between groups. The pooled random-effects estimate suggested no clear average benefit (SMD=−0.11, 95% CI −0.26 to 0.05; *P*=.16), based on 740 participants in the intervention groups and 730 in the control groups. Heterogeneity was moderate (Q_15_=23.54; *P*=.07; tau=0.106; tau²=0.0113; *I*²=36.3%). The prediction interval was −0.37 to 0.15, suggesting that the true effect may vary across settings and may not be consistently beneficial in all contexts.

To explore potential sources of between-study heterogeneity, prespecified subgroup analyses were conducted according to intervention duration, depression instrument, and follow-up time point, with results presented in Figures S2-S4 in Multimedia Appendix 1. When stratified by intervention duration (Figure S2 in Multimedia Appendix 1), the pooled effect for interventions lasting 5‐12 weeks was SMD=−0.19 (95% CI −0.59 to 0.20), whereas interventions longer than 12 weeks showed a smaller pooled effect (SMD=−0.06, 95% CI −0.21 to 0.09). The test for subgroup differences did not indicate statistically significant effect modification by duration. Subgrouping by depression measure (Figure S3 in Multimedia Appendix 1) similarly yielded no evidence of differential effects across instruments (test for subgroup differences, *P*=.43), with pooled estimates spanning from SMD=−0.33 (HADS-D) to approximately null effects for CES-D. Finally, stratification by follow-up time point (Figure S4 in Multimedia Appendix 1) suggested that effects varied across assessment windows: estimates were small at 1-≤3 months (SMD= −0.21, 95% CI −0.52 to 0.09), but were more pronounced and statistically significant at >3-≤6 months (SMD= −0.31, 95% CI −0.57 to −0.04; *P*=.03). At >6 months, effects were again closer to null (SMD= −0.07, 95% CI −0.22 to 0.09). Overall, these subgroup analyses did not provide strong evidence that intervention duration or outcome instrument accounted for heterogeneity, whereas follow-up stratification suggested a potential time-dependent pattern, with a more favorable effect at >3-≤6 months than at earlier or later assessment windows.

Additionally, 3 studies (Burke et al [14], Coulter et al [18], and Hearn et al [22]) used the HADS-Anxiety subscale to evaluate anxiety outcomes (Figure 2B). The pooled random-effects estimate was imprecise and did not show clear evidence of benefit (MD=−0.76, 95% CI −4.88 to 3.35; *P*=.51), based on 76 participants in the intervention groups and 66 in the control groups. Heterogeneity was moderate (Q_2_=3.69; *P*=.16; tau=0.889; tau²=0.7896; *I*²=45.8%).

#### Health-Related Quality of Life

**Figure 3.**
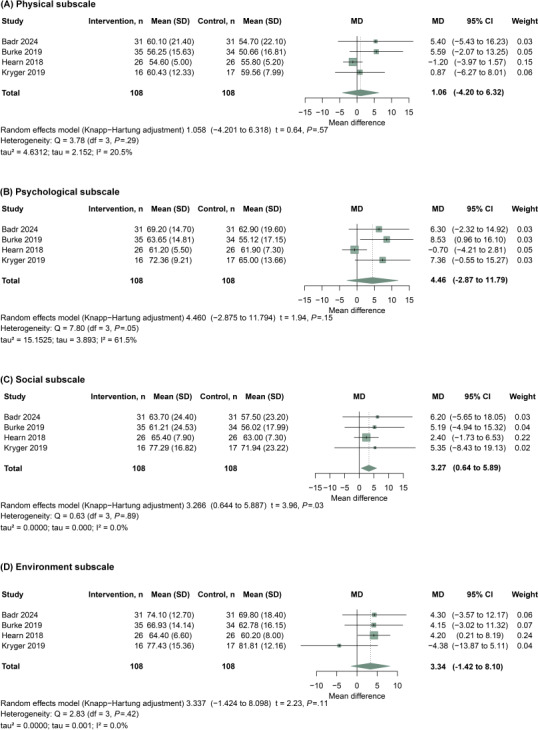
Meta-analysis of telehealth interventions on quality of life outcomes in individuals with spinal cord injury (SCI). Pooled effect sizes are presented for the 4 World Health Organization Quality of Life-BREF (WHOQOL-BREF) domains: physical, emotional, social, and environmental [11,14,22,29].

Four studies [11,14,22,29] reported WHOQOL-BREF domain scores and were included in the meta-analyses of health-related quality of life, covering the physical, psychological, social, and environmental domains (Figure 3). For each study, Figure 3 presents group-level summary statistics for the intervention and control groups (sample size, mean, and SD), together with the study-specific effect estimate and its 95% CI. Pooled effects were summarized as mean differences because all studies used the same instrument and domain scoring. Overall, telehealth interventions did not show clear evidence of benefit in the physical domain (MD=1.06, 95% CI −4.20 to 6.32; *P*=.57; Q_3_=3.78; *P*=.29; tau=4.6312; tau²=2.152; *I*²=20.5%) or the psychological domain (MD=4.46, 95% CI −2.87 to 11.79; *P*=.15; Q_3_=7.80; *P*=.05; tau=3.893; tau²=15.1525; *I*²=61.5%), whereas a statistically significant improvement was observed in the social domain (MD=3.27, 95% CI 0.64 to 5.89; *P*=.03; Q_3_=0.63; *P*=.89; tau=0.000; tau²=0.0000; *I*²=0.0%). Effects in the environmental domain were imprecise and did not show clear evidence of benefit (MD=3.34, 95% CI −1.42 to 8.10; *P*=.11; Q_3_=2.83; *P*=.42; tau=0.001; tau²=0.0000; *I*²=0.0%).

#### Sleep-Related Functioning

**Figure 4.**
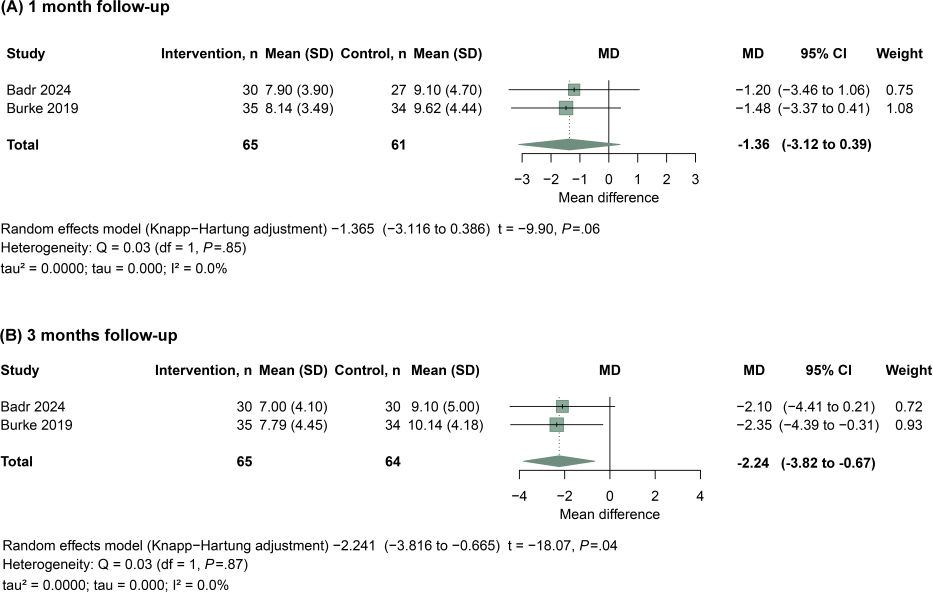
Meta-analysis of telehealth interventions on sleep quality in individuals with spinal cord injury (SCI). Sleep outcomes were assessed using the Pittsburgh Sleep Quality Index (PSQI). Studies were stratified by follow-up duration into short-term (1 month) and longer-term (3 months) subgroups [11,14].

This meta-analysis evaluated the impact of telehealth interventions on sleep-related functioning, using PSQI as the outcome measure (Figure 4). Studies were stratified into 2 subgroups based on follow-up duration. At 1 month (Figure 4A), the pooled random-effects estimate did not show clear evidence of benefit (MD= −1.36, 95% CI −3.12 to 0.39; *P*=.06), based on 65 participants in the intervention groups and 61 in the control groups, with no observed heterogeneity (Q_1_=0.03; *P*=.85; tau=0.000; tau²=0.0000; *I*²=0.0%); however, at 3 months (Figure 4B), telehealth interventions were associated with improved PSQI scores (MD= −2.24, 95% CI −3.82 to −0.67; *P*=.04), based on 65 participants in the intervention groups and 64 in the control groups, again with no observed heterogeneity (Q_1_=0.03; *P*=.87; tau=0.000; tau²=0.0000; *I*²=0.0%), indicating a statistically significant improvement in sleep-related functioning among patients receiving telehealth interventions.

#### Functional Independence and Rehabilitation Outcomes

**Figure 5.**
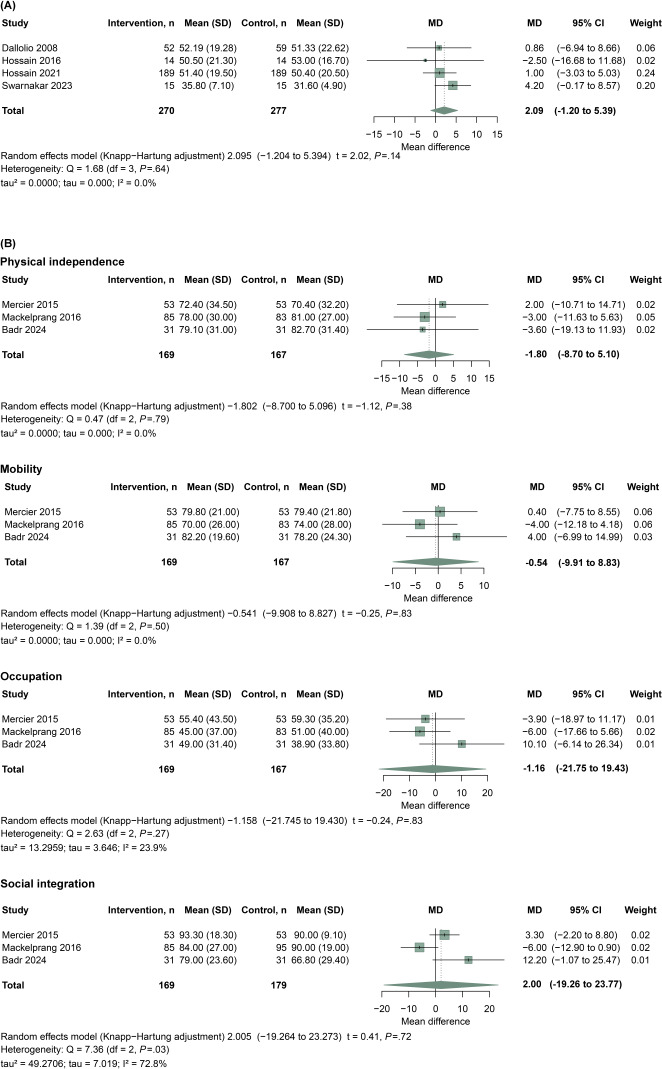
Meta-analysis of telehealth interventions on functional recovery in individuals with spinal cord injury (SCI). (A) Pooled analysis of 4 studies using the Spinal Cord Independence Measure (SCIM) to assess functional independence [19,23,24,40]. (B) Subgroup analysis using the Craig Handicap Assessment and Reporting Technique (CHART), evaluating 4 domains: physical independence, mobility, occupation, and social integration [11,35,36].

This meta-analysis included 4 studies [19,23,24,40] evaluating the impact of telehealth interventions on functional recovery in patients with SCI, using the SCIM scores as the outcome metric (Figure 5A). The pooled MD was 2.09 (95% CI −1.20 to 5.39; *P*=.14), indicating no clear average benefit. Between-study heterogeneity was negligible (Q_3_=1.68; *P*=.64; tau=0.00; tau²=0.00; *I*²=0.0%), suggesting that study findings were similar in direction and magnitude despite the absence of a statistically significant pooled effect. Between-study heterogeneity was negligible (Q_3_=1.68; *P*=.64; tau=0.00; tau²=0.00; *I*²=0.0%). To further explore domain-specific effects, functional outcomes were analyzed using CHART (Figure 5B), which includes physical independence, mobility, occupation, and social integration dimensions. In physical independence, telehealth interventions showed no statistically significant benefit (MD=−1.80, 95% CI −8.70 to 5.10; *P*=.38), with no observed heterogeneity (*I*^2^=0%). In the mobility domain, the effect was nonsignificant (MD=–0.54, 95% CI –9.91 to 8.83; *P*=.83). The pooled results across occupation and social integration dimensions also did not reach statistical significance (occupation, MD=–1.16, 95% CI −21.75 to 19.43; *P*=.83; social integration, MD=2.0, 95% CI −19.26 to 23.27, *P*=.72)

####  Pain Intensity

**Figure 6.**
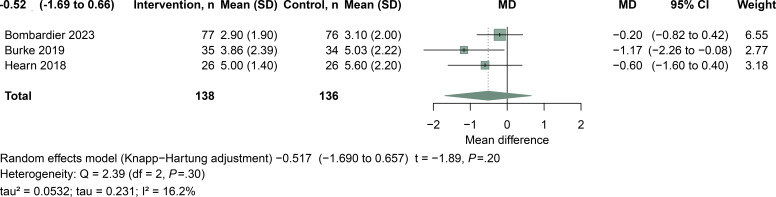
Meta-analysis of telehealth interventions on pain relief in individuals with spinal cord injury (SCI). Pain outcomes were assessed using the NRS [13,14,22].

This meta-analysis included 3 studies [13,14,22] that evaluated the effect of telehealth interventions on pain relief in patients with SCI, using the NRS as the outcome measure (Figure 6). The pooled random-effects estimate did not show clear evidence of benefit (MD=−0.52, 95% CI −1.69 to 0.66; *P*=.20), based on 138 participants in the intervention groups and 136 in the control groups. Between-study heterogeneity was low (Q_2_=2.39; *P*=.30; tau=0.231; tau²=0.0532; *I*²=16.2%).

### Certainty of Evidence

We assessed certainty of evidence for each critical outcome using GRADE and summarized results in a Summary of Findings table (Table S2 in Multimedia Appendix 1). Overall certainty ranged from high to low, with downgrading most commonly due to risk of bias and imprecision (small samples and wide confidence intervals), with detailed rationale provided in the Summary of Findings table footnotes. Evidence for depressive symptoms was of low certainty overall, although certainty varied by follow-up, including a high-certainty estimate for the >3-≤6 months subgroup and moderate or low certainty at other time windows. Evidence for anxiety was of low certainty, mainly limited by imprecision. For WHOQOL-BREF, certainty was moderate for the social relationships domain but low for the physical, psychological, and environment domains. For sleep quality assessed by PSQI, evidence at 1 month and 3 months was moderate certainty, downgraded primarily for imprecision because few trials reported sleep outcomes. For functional independence and participation, evidence was moderate for SCIM, while CHART domains ranged from moderate to low. For pain intensity (NRS), evidence was of low certainty.

## Discussion

### Principal Findings

Consistent with our objective to systematically review and meta-analyze the effects of telemedicine interventions on psychological health, health-related quality of life, sleep-related functioning, functional independence and rehabilitation outcomes, and pain intensity in patients with SCI, this review suggests that telemedicine may offer benefits for selected outcomes, but that these effects are domain-specific and time-dependent. The clearest signals of benefit were observed for the WHOQOL-BREF social domain and sleep quality at 3 months, findings that were supported by low between-study heterogeneity and moderate-certainty evidence. A more favorable effect on depressive symptoms was also observed in the >3-≤6 months follow-up subgroup, and although the certainty of evidence for this subgroup was high, this finding appears to reflect a time-specific benefit rather than a consistent overall effect across follow-up periods. By contrast, the overall pooled effect for depressive symptoms was not statistically significant, the prediction interval crossed the null, and certainty was low, suggesting that any overall benefit may be uncertain and may vary across settings. Evidence for anxiety, functional recovery, and pain intensity was also limited by imprecision, small sample sizes, lower-certainty evidence, and risk-of-bias concerns in a proportion of included trials, particularly related to deviations from intended interventions, outcome measurement, and selective reporting. Taken together, these findings suggest that telemedicine may serve as a useful complement to standard rehabilitation pathways in SCI, but that its effectiveness is likely to depend on the outcome targeted, intervention characteristics, follow-up window, and the strength and consistency of the underlying evidence across domains.

Telemedicine interventions may influence outcomes through two related but conceptually distinct pathways. First, remote delivery may be particularly suitable for individuals living with SCI by reducing barriers related to mobility, transportation, and geographic distance, thereby supporting continuity and timeliness of follow-up care [50-52]. Second, many telemedicine models embed enhanced care exposure, such as more frequent contacts, proactive monitoring, structured coaching, and facilitated self-management [53,54], which may improve outcomes irrespective of whether care is delivered remotely or in person. Because intervention dose and comparator intensity were variably reported and often differed across trials, the current evidence does not allow us to disentangle modality effects from care-intensity effects, and this should be considered when interpreting domain-specific benefits.

Psychological complications such as depression and anxiety are common complications following SCI [55]. A pooled analysis estimated the average prevalence of depression to be approximately 22% (95% CI 19%‐26%) [56]. Recent evidence syntheses highlight a substantial and persistent mental health burden after traumatic SCI, with depression being the most frequently studied condition [57]. Beyond prevalence, depressive symptoms have important clinical consequences. In a prospective follow-up study, higher depressive symptom burden was identified as a determinant of lower participation in inpatient rehabilitation [58]. Consistent with this, a large SCI Model Systems cohort study found that depression during inpatient rehabilitation was associated with poorer postdischarge outcomes at 1 year, including greater rehospitalization burden, worse pain severity, lower participation, and reduced life satisfaction, supporting the clinical value of early screening and timely intervention [59]. Positive affect is a crucial driver of self-management, whereas negative affect constitutes a significant barrier to self-care [60]. Our analysis further revealed that the most significant reduction in depressive symptoms occurred during the 3‐6 month follow-up period, whereas no significant effects were observed at 1‐3 months or beyond 6 months. We hypothesize that this may be attributed to the difficulty patients experience in accepting the reality of disability during the early stages of SCI, coupled with unstable treatment adherence. Over time, as patients gradually adapt and engage more actively with therapeutic measures, the benefits of telemedicine interventions become more evident during mid-term follow-up. Moreover, with the trend toward shorter hospital stays, patients with SCI now have fewer opportunities for inpatient rehabilitation and education, and higher adherence in the early postdischarge period may partially explain the absence of significant long-term differences [61,62]. Interestingly, one previous meta-analysis suggested no significant effects of telemedicine on depression at 3‐6 months (heterogeneity 59%) [8], whereas our findings demonstrated a significant benefit during the same period (heterogeneity 0%). This discrepancy highlights the need for additional high-quality studies to further clarify the actual impact of telemedicine on post-SCI depression. In addition, the contrast between the significant >3 and ≤6 months subgroup finding and the nonsignificant overall pooled effect indicates that any benefit for depressive symptoms should be interpreted as time-specific rather than as a consistent overall effect across follow-up periods, especially given the low certainty of the overall evidence. It is also important to note that different depression scales were used across the included studies, such as PHQ-9, HSCL-20B, BDI-II, CESD, DASS21-Depression, and HADS. In subgroup analyses restricted to HADS-based studies, the pooled estimate trended in the direction of improvement but remained statistically uncertain, highlighting limited power and the need for standardized assessment and reporting. Given that HADS has been widely used in medically ill populations and the depression subscale has supporting psychometric evidence [63], future trials using harmonized measures and clinically interpretable thresholds may help clarify whether the observed trends translate into consistent and clinically meaningful benefits.

With advances in medical care, the survival rate of patients with traumatic SCI has steadily increased [64]; however, this has been accompanied by long-term challenges such as mobility impairments, polypharmacy, bladder and bowel dysfunction, and restrictions in activities of daily living, all of which markedly reduce quality of life [65]. Observational studies using the WHOQOL-BREF consistently show substantially lower scores across domains in people with SCI compared with reference populations. In some cohorts, the psychological domain is among the lowest-scoring domains, underscoring the multidimensional and persistent burden of reduced quality of life in this population [66-68]. In our synthesis, telemedicine showed the most consistent benefit in the WHOQOL-BREF social domain at short-term follow-up, whereas evidence for the physical, psychological, and environmental domains remained uncertain and was supported by lower-certainty evidence. The social dimension encompasses interpersonal relationships, sexual life, and social support from friends, where telemedicine also showed favorable effects. In line with our findings, a previous meta-analysis reported that telemedicine interventions improved quality of life at 3 months, whereas the between-group difference disappeared at 12 months. However, given the limited sample size (only two studies), the representativeness of these results remains debatable [8]. Future trials should therefore report domain-level trajectories with adequate follow-up, document intervention intensity and engagement, and include clinically interpretable benchmarks to clarify whether early domain-specific gains translate into sustained improvements in broader quality-of-life outcomes.

Sleep disturbances are common after SCI and can exacerbate comorbidities that directly shape health-related quality of life, particularly mood, pain, and daytime functioning [69,70]. Recent syntheses indicate a substantial burden of insomnia symptoms in SCI and emphasize the need for routine sleep assessment across the continuum of care [71]. Among the studies included in our review, only two systematically assessed the effects of telemedicine using the PSQI. Our findings suggest that telemedicine had a limited impact at 1 month but more favorable effects at 3 months, although this interpretation should remain cautious because sleep outcomes were informed by only a small number of trials despite moderate-certainty evidence. The observed pattern of minimal short-term change with more favorable effects after sustained intervention is clinically plausible, as sleep improvements often require iterative adjustment and adherence over time. Consistent with this, a 12-week mHealth program incorporating multiple behavior-change strategies showed a favorable trend toward improved PSQI scores [72], and an earlier systematic review reported that telecounseling may improve sleep difficulties as part of broader comorbidity management, although the evidence base was small [54]. Until a larger and more methodologically consistent evidence base is available, conclusions regarding sleep benefits should remain cautious, and emphasis should be placed on standardizing definitions, follow-up windows, and reporting across trials.

Concerning functional independence, we did not observe significant improvements in pooled SCIM or CHART outcomes, and the overall evidence remained uncertain despite negligible heterogeneity for SCIM and moderate certainty for that outcome. Nonetheless, several individual trials provide signals that may help explain which intervention components are more likely to support functional gains. Nonetheless, individual trials provide signals that warrant interpretation. Swarnakar et al [40] reported substantial improvements in SCIM self-care and mobility domains after 8 weeks of telemedicine, with greater overall gains than controls. Similarly, Dallolio et al [19] found that 6 months of intervention significantly improved Functional Independence Measure (FIM) scores, particularly in grooming, dressing, and transfers, consistent with trends observed in SCIM. In contrast, Hossain et al [24] did not observe improvements in SCIM, which may reflect the intervention’s focus on complications such as pressure ulcers, urinary tract infections, incontinence, depression, and respiratory conditions rather than targeted functional training. Moreover, the absence of patient selection based on risk profiles or proactive help-seeking behaviors may have diluted the intervention’s impact [35]. Given that rehabilitation services in specialized centers are time-limited, telemedicine offers a valuable opportunity to extend long-term rehabilitation and professional guidance, particularly during the COVID-19 pandemic, when maintaining continuity of care remained crucial [40]. Taken together, these findings suggest that functional gains from telemedicine are more likely when interventions include clearly specified functional training components with sufficient intensity and when they are delivered to patients most likely to benefit.

Chronic pain is another common and debilitating consequence of SCI, with profound effects on daily functioning, mental health, and quality of life [73,74]. Oral pharmacological treatment for neuropathic pain after SCI is often inadequate, commonly resulting in only about a 20%‐30% reduction in pain intensity [75], while neuromodulation approaches (eg, transcranial direct current stimulation, transcranial magnetic stimulation) face limitations related to uncertain efficacy and accessibility [76]. Cognitive behavioral therapy–based pain management programs (CBT-PMP) have shown efficacy in improving mood, pain, and daily activities among individuals with SCI [77]. However, their broader implementation is constrained by mobility challenges and the need for specialized expertise [14]. In this context, telemedicine offers a practical route to extend access to structured psychological pain management. A Cochrane review of remotely delivered psychological therapies for chronic pain concluded that internet-delivered CBT can produce small improvements in pain intensity and functional disability, although benefits were not consistently maintained at follow-up, and evidence certainty varied across outcomes [78]. Telemedicine, particularly when integrated with CBT-PMP, is a promising approach to overcoming these barriers. Previous meta-analyses have demonstrated that internet-based psychological interventions can significantly alleviate symptoms in patients with chronic pain [79] and chronic diseases [80]. Although our review did not find significant improvements in SCI-related pain, the available trials suggested a possible benefit, but the evidence remained limited by small sample sizes, low certainty, and imprecision. Larger, multicenter RCTs with sufficient power are needed to clarify the true efficacy of telemedicine for pain management in SCI.

### Limitations

Several limitations should be considered when interpreting these findings. First, the number of included studies was relatively small, and some outcomes, such as sleep and pain, were reported in only a few trials, limiting precision and generalizability. Second, clinical and methodological heterogeneity was substantial, including variation in intervention modality, content, intensity, follow-up duration, and outcome measures, which likely contributed to imprecision and limited inference about which telemedicine approaches work best for specific outcomes. Third, many trials were underpowered and had relatively short follow-up, which constrains the assessment of durability and may underestimate longer-term effects that depend on sustained engagement. In addition, even within RCTs, differential uptake and engagement may occur after randomization. Participants with greater digital literacy, technology access, or comfort with telehealth may be more likely to adhere to and benefit from telemedicine interventions, whereas those with lower access or skills may disengage, which could introduce bias through deviations from intended interventions and differential attrition. Few trials reported baseline digital literacy or technology access, limiting our ability to assess effect modification by these factors and potentially constraining generalizability to settings with limited digital resources. Finally, subgroup analyses by delivery modality were limited, as most interventions relied primarily on telephone contacts, whereas emerging approaches such as virtual coaching, synchronous video, and app-supported monitoring may differ in effectiveness across domains.

### Conclusion

This systematic review and meta-analysis offer an up-to-date, domain-based synthesis of randomized evidence on telemedicine interventions for SCI. Compared with earlier reviews, it incorporates additional recent trials and evaluates five prespecified outcome domains, enabling a more granular assessment of where evidence is most consistent and where uncertainty remains. Overall, telemedicine may be associated with improvements in selected patient-important outcomes, with the most consistent signals observed for social aspects of health-related quality of life and sleep-related functioning after sustained intervention exposure, whereas effects on depression, functional independence, and pain are less consistent and remain uncertain in several analyses. In real-world SCI care, these findings support telemedicine as a pragmatic adjunct to standard rehabilitation and follow-up, particularly for extending continuity and access for individuals facing mobility, geographic, or resource barriers. Future adequately powered randomized trials should standardize outcomes and follow-up, report intervention dose and engagement, and evaluate which telemedicine modalities and components work best for specific outcomes and patient subgroups to inform domain-specific, personalized tele-rehabilitation strategies.

## Supplementary material

10.2196/87088 Multimedia Appendix 1 Database search strategies and summary of findings for telemedicine outcomes in spinal cord injury.

10.2196/87088 Checklist 1 PRISMA (Preferred Reporting Items for Systematic Reviews and Meta-Analyses) 2020 expanded checklist.

## abbreviations

BDI: Beck Depression Inventory
CBT-PMP: Cognitive behavioral therapy–based pain management program
CENTRAL: Cochrane Central Register of Controlled Trials
CES-D: Center for Epidemiologic Studies Depression Scale
CHART: Craig Handicap Assessment and Reporting Technique
EQ-5D: EuroQol Five Dimensions Questionnaire
ESS: Epworth Sleepiness Scale
FIM: Functional Independence Measure
GRADE: Grading of Recommendations Assessment, Development and Evaluation
HADS: Hospital Anxiety and Depression Scale
MD: mean difference
NRS: Numerical Rating Scale
PHQ-9: Patient Health Questionnaire-9
PRISMA: Preferred Reporting Items for Systematic Reviews and Meta-Analyses
PRISMA-S: Preferred Reporting Items for Systematic Reviews and Meta-Analyses–Search
PSQI: Pittsburgh Sleep Quality Index
PUSH: Pressure Ulcer Scale for Healing
RCT: randomized controlled trial
RoB 2: Risk of Bias 2
SCI: spinal cord injury
SCIM: Spinal Cord Independence Measure
SF-36: Short Form Health Survey-36
SMD: standardized mean difference
WHOQOL-BREF: World Health Organization Quality of Life-BREF
