# Supplementary material for: Multidimensional Effects of Telemedicine on Patients With Spinal Cord Injury: Systematic Review and Meta-Analysis of Randomized Controlled Trials
Source: J Med Internet Res. 2026 May 6;28:e87088. doi: 10.2196/87088 (PMC13148336; doi:10.2196/87088)
Supplement: Multimedia Appendix 1 [file jmir-v28-e87088-s001.docx]

Table S1. Detailed Search Strategies Across Databases.

| **PubMed** |
| --- |
| #1 ( "Spinal Cord Injuries"[Mesh] OR spinal cord injur*[tiab] OR spinal cord trauma*[tiab] OR traumatic myelopath*[tiab] OR paraplegi*[tiab] OR tetraplegi*[tiab] OR myelopath* post-traumatic[tiab] OR spinal cord contusion*[tiab] OR spinal cord laceration*[tiab] OR spinal cord transection*[tiab] ) 96,494 #2 ( telerehabilitation[tiab] OR tele-rehabilitation[tiab] OR telerehab*[tiab] OR tele-rehab*[tiab] OR "tele rehabilitation"[tiab] OR "remote rehabilitation"[tiab] OR "virtual rehabilitation"[tiab] OR ( ( "Telemedicine"[Mesh] OR telehealth[tiab] OR tele-health[tiab] OR telemedicine[tiab] OR tele-medicine[tiab] OR telecare[tiab] OR tele-care[tiab] OR teleconsult*[tiab] OR tele-consult*[tiab] OR "video consultation"[tiab] OR videoconferenc*[tiab] OR video-conferenc*[tiab] OR "video conferencing"[tiab] OR teleconferenc*[tiab] OR tele-conferenc*[tiab] OR ehealth[tiab] OR e-health[tiab] OR "electronic health"[tiab] OR mhealth[tiab] OR "mobile health"[tiab] OR "digital health"[tiab] OR "internet-based"[tiab] OR "web-based"[tiab] OR smartphone*[tiab] OR app[tiab] OR apps[tiab] OR telephone*[tiab] OR online[tiab] OR "online program*"[tiab] OR "online intervention*"[tiab] OR "online rehabilitation"[tiab] ) AND ( "Rehabilitation"[Mesh] OR "Physical Therapy Modalities"[Mesh] OR rehabilitat*[tiab] OR physiotherap*[tiab] OR "physical therap*"[tiab] OR "exercise therap*"[tiab] OR exercis*[tiab] OR training[tiab] ) ) )  #3 (randomized controlled trial[pt] OR controlled clinical trial[pt] OR randomized[tiab] OR placebo[tiab] OR clinical trials as topic[mesh:noexp] OR randomly[tiab] OR trial[ti] NOT (animals[mh] NOT humans [mh])) 1,638,881 97,639 #4 #1 and #2 and #3 101 |
| **Web of science** |
| # Searches:  1: TS=("spinal cord injur*" OR "spinal cord trauma*" OR "traumatic myelopath*" OR paraplegi* OR tetraplegi* OR ("myelopath*" NEAR/2 post-traumatic) OR "spinal cord contusion*" OR "spinal cord laceration*" OR "spinal cord transection*") Results: 91728  2: TS=(telerehabilitation OR tele-rehabilitation OR telerehab* OR tele-rehab* OR "tele rehabilitation" OR "remote rehabilitation" OR "virtual rehabilitation") Results: 6131  3: TS=(telehealth OR tele-health OR telemedicine OR tele-medicine OR telecare OR tele-care OR teleconsult* OR tele-consult* OR "video consultation" OR videoconferenc* OR video-conferenc* OR "video conferencing" OR teleconferenc* OR tele-conferenc* OR ehealth OR e-health OR "electronic health" OR mhealth OR "mobile health" OR "digital health" OR "internet-based" OR "web-based" OR smartphone* OR app OR apps OR telephone* OR online OR "online program*" OR "online intervention*" OR "online rehabilitation") Results: 1297502  4: TS=(rehabilitat* OR physiotherap* OR "physical therap*" OR "exercise therap*" OR exercis* OR training) Results: 2778595  5: #2 OR (#3 AND #4) Results: 162483  6: TS=(random* OR trial OR placebo OR "controlled trial" OR "randomized controlled trial" OR "randomised controlled trial") Results: 4233848  7: #1 AND #5 AND #6 Results: 249 |
| **Embase Ovid MEDLINE(R) ALL** |
| Embase <1974 to 2026 February 24>  Ovid MEDLINE(R) ALL <1946 to February 24, 2026>  1 exp spinal cord injury/ 166723  2 (spinal cord injur* or spinal cord trauma* or traumatic myelopath* or paraplegi* or tetraplegi* or (myelopath* adj2 post-traumatic) or spinal cord contusion* or spinal cord laceration* or spinal cord transection*).ti,ab. 169651  3 1 or 2 230050  4 (telerehabilitation or tele-rehabilitation or telerehab* or tele-rehab* or "tele rehabilitation" or "remote rehabilitation" or "virtual rehabilitation").ti,ab. 8362  5 exp telemedicine/ 152002  6 (telehealth or tele-health or telemedicine or tele-medicine or telecare or tele-care or teleconsult* or tele-consult* or "video consultation" or videoconferenc* or video-conferenc* or "video conferencing" or teleconferenc* or tele-conferenc* or ehealth or e-health or "electronic health" or mhealth or "mobile health" or "digital health" or "internet-based" or "web-based" or smartphone* or app or apps or telephone* or online or "online program*" or "online intervention*" or "online rehabilitation").ti,ab. 1430604  7 5 or 6 1482537  8 exp rehabilitation/ 1006321  9 exp physiotherapy/ 140090  10 exp exercise therapy/ 202976  11 (rehabilitat* or physiotherap* or "physical therap*" or "exercise therap*" or exercis* or training).ti,ab. 3027066  12 8 or 9 or 10 or 11 3706050  13 4 or (7 and 12) 242221  14 randomized controlled trial/ or randomization/ or double blind procedure/ or single blind procedure/ or crossover procedure/ 2078337  15 (random* or trial or placebo or "double blind" or "single blind").ti,ab. 5630088  16 14 or 15 5936844  17 animal/ not human/ 6673333  18 16 not 17 5632364  19 3 and 13 and 18 418 |
| **EBM Reviews - Cochrane Central Register of Controlled Trials** |
| EBM Reviews - Cochrane Central Register of Controlled Trials <January 2026>  1 exp spinal cord injury/ 2632  2 (spinal cord injur* or spinal cord trauma* or traumatic myelopath* or paraplegi* or tetraplegi* or (myelopath* adj2 post-traumatic) or spinal cord contusion* or spinal cord laceration* or spinal cord transection*).ti,ab. 5036  3 1 or 2 5434  4 (telerehabilitation or tele-rehabilitation or telerehab* or tele-rehab* or "tele rehabilitation" or "remote rehabilitation" or "virtual rehabilitation").ti,ab. 2092  5 exp telemedicine/ 5878  6 (telehealth or tele-health or telemedicine or tele-medicine or telecare or tele-care or teleconsult* or tele-consult* or "video consultation" or videoconferenc* or video-conferenc* or "video conferencing" or teleconferenc* or tele-conferenc* or ehealth or e-health or "electronic health" or mhealth or "mobile health" or "digital health" or "internet-based" or "web-based" or smartphone* or app or apps or telephone* or online or "online program*" or "online intervention*" or "online rehabilitation").ti,ab. 94657  7 5 or 6 95850  8 exp rehabilitation/ 58398  9 exp rehabilitation/ 58398  10 exp exercise therapy/ 23972  11 (rehabilitat* or physiotherap* or "physical therap*" or "exercise therap*" or exercis* or training).ti,ab. 280903  12 8 or 9 or 10 or 11 304263  13 4 or (7 and 12) 28798  14 randomized controlled trial/ or randomization/ or double blind procedure/ or single blind procedure/ or crossover procedure/ 25263  15 (random* or trial or placebo or "double blind" or "single blind").ti,ab. 1605729  16 14 or 15 1609048  17 animal/ not human/ 17175  18 16 not 17 1596835  19 3 and 13 and 18 168 |

*Table S2*. Summary of Findings: Effects of Telemedicine on Psychological Health, Quality of Life, Sleep, Function, and Pain in Spinal Cord Injury.

| **Certainty assessment** | | | | | | | **№ of patients** | | **Effect** | | **Certainty** | **Importance** |
| --- | --- | --- | --- | --- | --- | --- | --- | --- | --- | --- | --- | --- |
| **№ of studies** | **Study design** | **Risk of bias** | **Inconsistency** | **Indirectness** | **Imprecision** | **Other considerations** | **Telemedicine** | **usual care** | **Relative (95% CI)** | **Absolute (95% CI)** |  |  |
| **Mental health-Depression** | | | | | | | | | | | | |
| 15 | randomised trials | serious^a^ | not serious^b^ | not serious^c^ | serious^d^ | none | 715 | 703 | - | SMD **0.12 SD lower** (0.28 lower to 0.04 higher) | ⨁⨁◯◯ Low^a,b,c,d^ | CRITICAL |
| **Mental health-Depression(1 month follow-up）** | | | | | | | | | | | | |
| 2 | randomised trials | not serious^a^ | not serious^b^ | not serious^c^ | serious^d^ | none | 57 | 53 | - | SMD **1.01 SD lower** (8.17 lower to 6.16 higher) | ⨁⨁⨁◯ Moderate^a,b,c,d^ | CRITICAL |
| **Mental health-Depression(1 to ≤ 3 months follow-up）** | | | | | | | | | | | | |
| 9 | randomised trials | serious^a^ | not serious | not serious | serious^d^ | none | 272 | 266 | - | SMD **0.21 SD lower** (0.52 lower to 0.09 higher) | ⨁⨁◯◯ Low^a,d^ | CRITICAL |
| **Mental health-Depression(＞3 to ≤ 6 months follow-up）** | | | | | | | | | | | | |
| 4 | randomised trials | not serious^a^ | not serious | not serious | not serious | none | 195 | 195 | - | SMD **0.31 SD lower** (0.57 lower to 0.04 lower) | ⨁⨁⨁⨁ High^a^ | CRITICAL |
| **Mental health-Depression(＞6 months follow-up）** | | | | | | | | | | | | |
| 6 | randomised trials | not serious^a^ | not serious | not serious | serious^d^ | none | 430 | 430 | - | SMD **0.24 SD lower** (0.22 lower to 0.09 higher) | ⨁⨁⨁◯ Moderate^a,d^ | CRITICAL |
| **Mental health-Anxiety** | | | | | | | | | | | | |
| 3 | randomised trials | serious^a^ | not serious | not serious | serious^d^ | none | 76 | 66 | - | MD **0.76 lower** (4.88 lower to 3.35 higher) | ⨁⨁◯◯ Low^a,d^ | CRITICAL |
| **WHOQOL-BREF physical** | | | | | | | | | | | | |
| 4 | randomised trials | serious^a^ | not serious | not serious | serious | none | 108 | 108 | - | MD **1.06 higher** (4.2 lower to 6.32 higher) | ⨁⨁◯◯ Low^a^ | CRITICAL |
| **WHOQOL-BREF psychological** | | | | | | | | | | | | |
| 4 | randomised trials | serious^a^ | not serious | not serious | serious^d^ | none | 108 | 108 | - | MD **4.46 higher** (2.87 lower to 11.79 higher) | ⨁⨁◯◯ Low^a,d^ | CRITICAL |
| **WHOQOL-BREF social** | | | | | | | | | | | | |
| 4 | randomised trials | serious^a^ | not serious | not serious | not serious | none | 108 | 108 | - | MD **3.27 higher** (0.64 higher to 5.89 higher) | ⨁⨁⨁◯ Moderate^a^ | CRITICAL |
| **WHOQOL-BREF environment** | | | | | | | | | | | | |
| 4 | randomised trials | serious^a^ | not serious | not serious | serious^d^ | none | 108 | 108 | - | MD **3.34 higher** (1.42 lower to 8.1 higher) | ⨁⨁◯◯ Low^a,d^ | CRITICAL |
| **PSQI(1 month follow-up）** | | | | | | | | | | | | |
| 2 | randomised trials | not serious | not serious | not serious | serious^d^ | none | 65 | 61 | - | MD **1.36 lower** (3.12 lower to 0.39 higher) | ⨁⨁⨁◯ Moderate^d^ | CRITICAL |
| **PSQI(3 month follow-up）** | | | | | | | | | | | | |
| 2 | randomised trials | not serious | not serious | not serious | serious^d^ | none | 65 | 64 | - | MD **2.24 lower** (3.82 lower to 0.67 higher) | ⨁⨁⨁◯ Moderate^d^ | CRITICAL |
| **SCIM** | | | | | | | | | | | | |
| 4 | randomised trials | not serious | not serious | not serious | serious^d^ | none | 270 | 277 | - | MD **2.09 higher** (1.2 lower to 5.39 higher) | ⨁⨁⨁◯ Moderate^d^ | CRITICAL |
| **CHART-physical independence** | | | | | | | | | | | | |
| 3 | randomised trials | not serious | not serious | not serious | serious^d^ | none | 169 | 167 | - | MD **1.8 lower** (8.7 lower to 5.1 higher) | ⨁⨁⨁◯ Moderate^d^ | CRITICAL |
| **CHART-mobility** | | | | | | | | | | | | |
| 3 | randomised trials | not serious | not serious | not serious | serious^d^ | none | 169 | 167 | - | MD **0.54 lower** (9.91 lower to 8.83 higher) | ⨁⨁⨁◯ Moderate^d^ | CRITICAL |
| **CHART-occupation** | | | | | | | | | | | | |
| 3 | randomised trials | not serious | not serious | not serious | very serious^d^ | none | 169 | 167 | - | MD **1.16 lower** (21.75 lower to 19.43 higher) | ⨁⨁◯◯ Low^d^ | CRITICAL |
| **CHART-social integration** | | | | | | | | | | | | |
| 3 | randomised trials | not serious | not serious | not serious | very serious^d^ | none | 169 | 167 | - | MD **2 higher** (19.26 lower to 23.27 higher) | ⨁⨁◯◯ Low^d^ | CRITICAL |
| **NRS** | | | | | | | | | | | | |
| 3 | randomised trials | serious^a^ | not serious | not serious | serious^d^ | none | 138 | 136 | - | MD **0.52 lower** (1.69 lower to 0.66 higher) | ⨁⨁◯◯ Low^a,d^ | CRITICAL |

**CI:** confidence interval; **MD:** mean difference; **SMD:** standardised mean difference

#### Explanations

a. Not serious: Most studies, and ideally most meta-analysis weight, are at low risk of bias, with no major concerns in key domains. Serious (downgrade 1): Many studies are rated some concerns or high risk, or a meaningful share of the meta-analysis weight comes from high-risk studies, and bias is likely to affect the estimate. Very serious (downgrade 2): Most studies, or most meta-analysis weight, are at high risk of bias, and critical limitations are very likely to change the estimate.

b. Not serious: Low heterogeneity and effects largely consistent, typically I² ≤ 50%. Serious (downgrade 1): Moderate heterogeneity or variable effects not explained, typically I² 50–75%. Very serious (downgrade 2): Substantial heterogeneity or conflicting effects not explained, typically I² ≥ 75%.

c. Not serious: Evidence directly matches the target PICO. Serious (downgrade 1): Important differences in population, intervention, comparator, outcomes, or setting. Very serious (downgrade 2): Most evidence does not address the target PICO.

d. Not serious: 95% CI is sufficiently narrow to support a clear conclusion. Serious (downgrade 1): 95% CI crosses no effect or includes both no important effect and clinically important benefit. Very serious (downgrade 2): Very wide 95% CI, often with few studies or small samples, spanning important benefit and no effect or harm


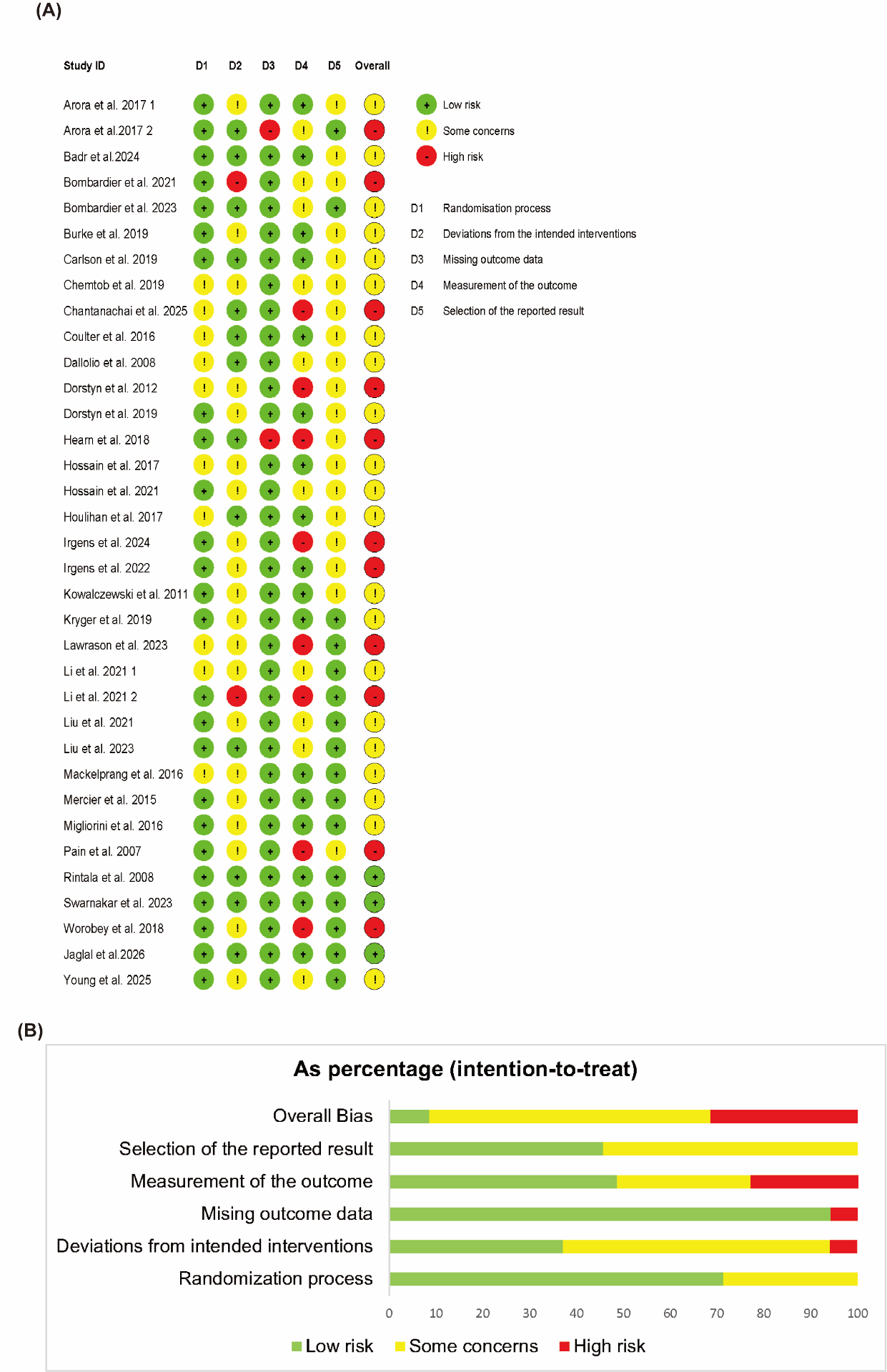


Figure S1. Risk of Bias Assessment of Included Studies Using ROB 2.0 Tool. (A) Traffic light plot showing domain-level and overall risk of bias assessments for each individual study. Each row represents a study, and each column corresponds to a specific domain or the overall judgment. Color coding: green = low risk, yellow = some concerns, red = high risk. (B) Proportional distribution of risk of bias judgments across five domains for all included studies. This summary plot illustrates the percentage of studies rated as “Low risk,” “Some concerns,” or “High risk” in each domain, providing an overview of methodological quality [9-43].


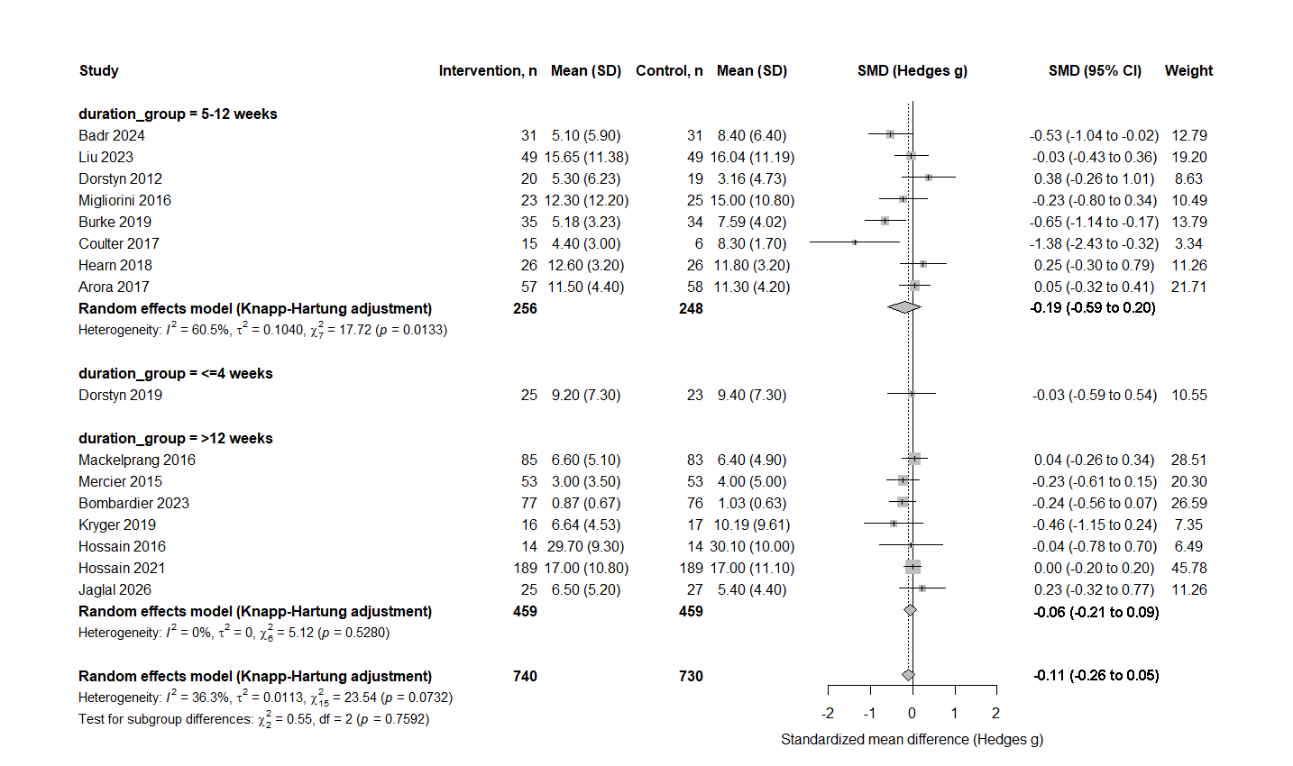


Figure S2. Depression subgroup analysis by intervention duration. Studies are grouped by intervention duration (≤4 weeks, 5–12 weeks, and >12 weeks). Negative values favor the intervention [10, 11, 13, 14, 18, 20, 22-24, 29, 34-37, 42].

11
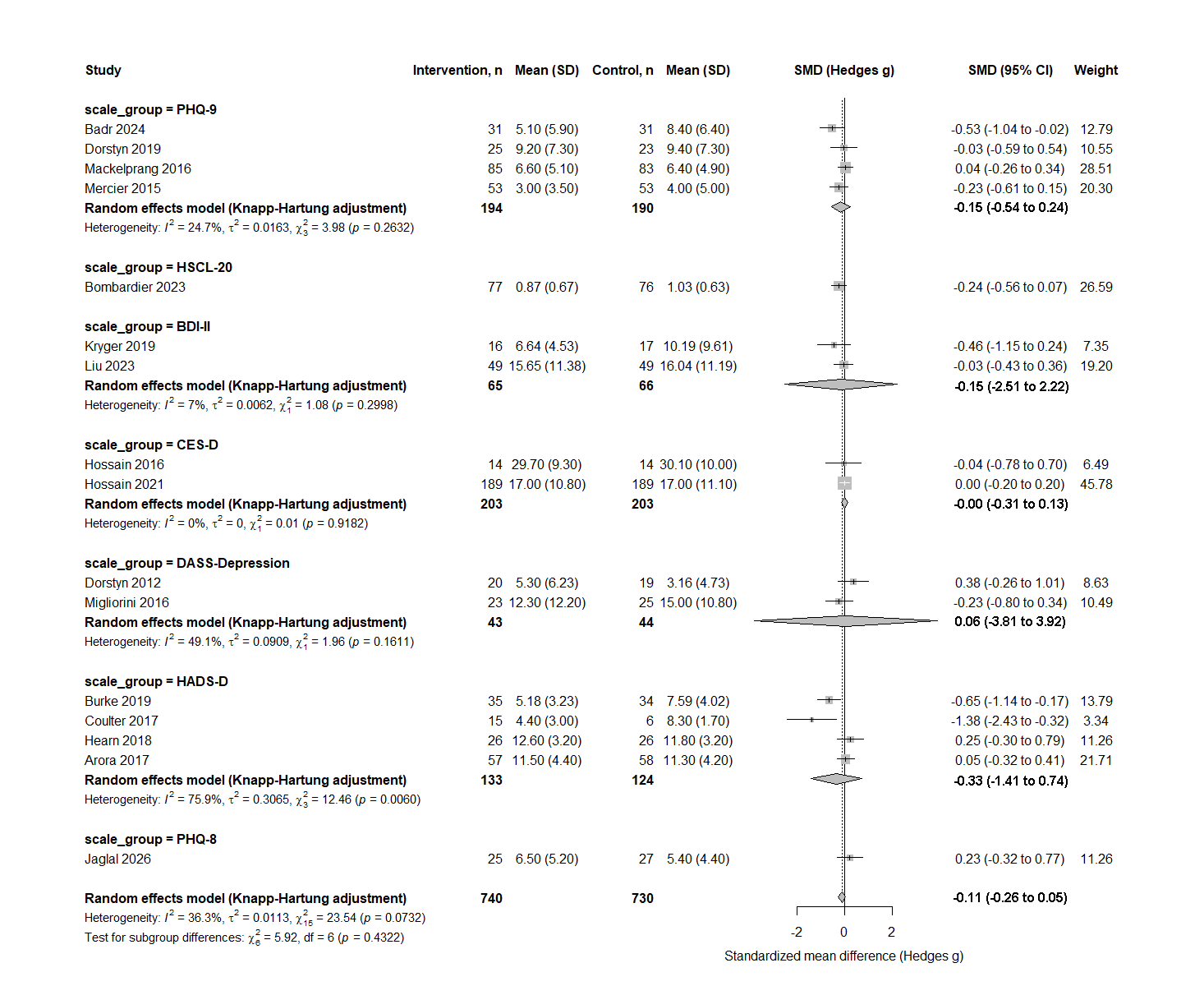


Figure S3. Depression subgroup analysis by outcome instrument. Studies are grouped by depression measure (PHQ-9, BDI-II, CES-D, DASS-Depression, and HADS-D) [10,11,13,14,18,20,22-24,29,30,35-37,39,42].


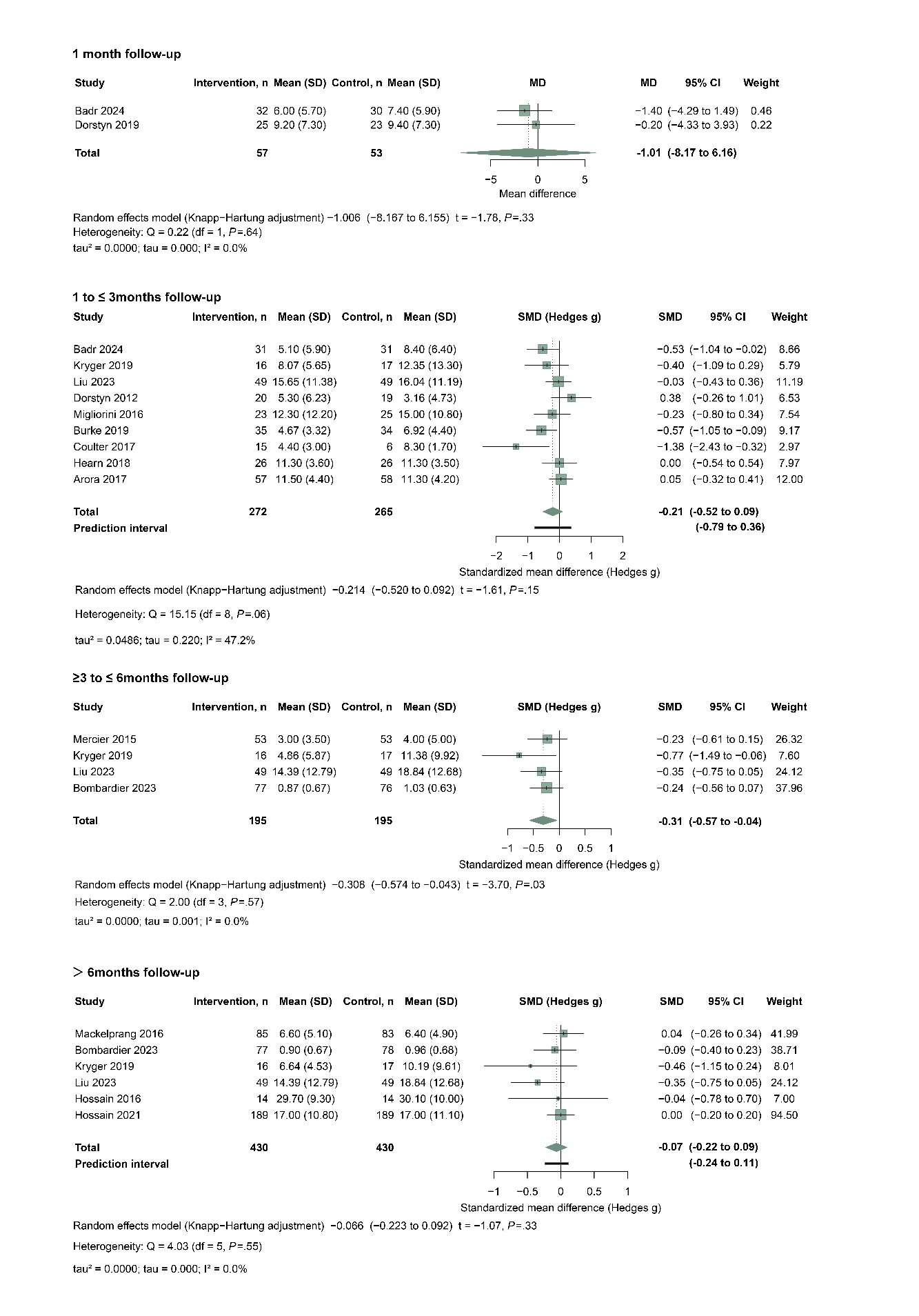


Figure S4. Depression subgroup analysis by follow-up time point. Studies are grouped by follow-up time point (1 month, 1 to ≤3 months, >3 to ≤6 months, and >6 months) [10,11,13-15,18-26,28-43].

References:

9. Arora M, Harvey LA, Glinsky JV, et al. Cost-effectiveness analysis of telephone-based support for the management of pressure ulcers in people with spinal cord injury in India and Bangladesh. Spinal Cord. Dec 2017;55(12):1071-1078. [doi: ] [Medline: 28809389]

10. Arora M, Harvey LA, Glinsky JV, et al. Telephone-based management of pressure ulcers in people with spinal cord injury in low- and middle-income countries: a randomised controlled trial. Spinal Cord. Feb 2017;55(2):141-147. [doi: ] [Medline: 27995939]

11. Badr MS, Martin JL, Sankari A, et al. Intensive support does not improve positive-airway pressure use in spinal cord injury/disease: a randomized clinical trial. Sleep. May 10, 2024;47(5):zsae044. [doi: ] [Medline: 38422375]

12. Bombardier CH, Dyer JR, Burns P, et al. A tele-health intervention to increase physical fitness in people with spinal cord injury and cardiometabolic disease or risk factors: a pilot randomized controlled trial. Spinal Cord. Jan 2021;59(1):63-73. [doi: ] [Medline: 32694748]

13. Bombardier CH, Fann JR, Ehde DM, et al. Collaborative CARE versus usual CARE to improve quality of life, pain, depression, and physical activity in outpatients with spinal cord injury: The SCI-CARE randomized controlled clinical trial. J Neurotrauma. Dec 2023;40(23-24):2667-2679. [doi: ] [Medline: 37597201]

14. Burke D, Lennon O, Blake C, et al. An internet-delivered cognitive behavioural therapy pain management programme for spinal cord injury pain: a randomized controlled trial. Eur J Pain. Aug 2019;23(7):1264-1282. [doi: ] [Medline: 31002442]

15. Carlson M, Vigen CLP, Rubayi S, et al. Lifestyle intervention for adults with spinal cord injury: results of the USC-RLANRC pressure ulcer prevention study. J Spinal Cord Med. Jan 2019;42(1):2-19. [doi: ] [Medline: 28414254]

16. Chantanachai T, Apiworajirawit I, Klamruen P, et al. Tele-rehabilitation using transcranial direct current stimulation combined with exercise in people with spinal cord injury: a randomized controlled trial. J Rehabil Med. May 7, 2025;57:jrm42353. [doi: ] [Medline: 40331505]

17. Chemtob K, Rocchi M, Arbour-Nicitopoulos K, Kairy D, Fillion B, Sweet SN. Using tele-health to enhance motivation, leisure time physical activity, and quality of life in adults with spinal cord injury: a self-determination theory-based pilot randomized control trial. Psychol Sport Exerc. Jul 2019;43:243-252. [doi: ]

18. Coulter EH, McLean AN, Hasler JP, Allan DB, McFadyen A, Paul L. The effectiveness and satisfaction of web-based physiotherapy in people with spinal cord injury: a pilot randomised controlled trial. Spinal Cord. Apr 2017;55(4):383-389. [doi: ] [Medline: 27596027]

19. Dallolio L, Menarini M, China S, et al. Functional and clinical outcomes of telemedicine in patients with spinal cord injury. Arch Phys Med Rehabil. Dec 2008;89(12):2332-2341. [doi: ] [Medline: 19061746]

20. Dorstyn D, Mathias J, Denson L, Robertson M. Effectiveness of telephone counseling in managing psychological outcomes after spinal cord injury: a preliminary study. Arch Phys Med Rehabil. Nov 2012;93(11):2100-2108. [doi: ] [Medline: 22705237]

21. Dorstyn D, Roberts R, Murphy G, et al. Work and SCI: a pilot randomized controlled study of an online resource for job-seekers with spinal cord dysfunction. Spinal Cord. Mar 2019;57(3):221-228. [doi: ] [Medline: 30262878]

22. Hearn JH, Finlay KA. Internet-delivered mindfulness for people with depression and chronic pain following spinal cord injury: a randomized, controlled feasibility trial. Spinal Cord. Aug 2018;56(8):750-761. [doi: ] [Medline: 29581519]

23. Hossain MS, Harvey LA, Rahman MA, et al. A pilot randomised trial of community-based care following discharge from hospital with a recent spinal cord injury in Bangladesh. Clin Rehabil. Jun 2017;31(6):781-789. [doi: ] [Medline: 27311454]

24. Hossain MS, Harvey LA, Islam MS, et al. A community-based intervention to prevent serious complications and death 2 years after discharge in people with spinal cord injury in Bangladesh (CIVIC): a randomised trial. Spinal Cord. Jun 2021;59(6):649-658. [doi: ] [Medline: 32917948]

25. Houlihan BV, Brody M, Everhart-Skeels S, et al. Randomized trial of a peer-led, telephone-based empowerment intervention for persons with chronic spinal cord injury improves health self-management. Arch Phys Med Rehabil. Jun 2017;98(6):1067-1076. [doi: ] [Medline: 28284835]

26. Irgens I, Midelfart-Hoff J, Jelnes R, et al. Videoconferencing in pressure injury: randomized controlled telemedicine trial in patients with spinal cord injury. JMIR Form Res. Apr 19, 2022;6(4):e27692. [doi: ] [Medline: 35438645]

27. Irgens I, Kleven L, Midelfart-Hoff J, et al. Cost-utility analysis and impact on the environment of videoconference in pressure injury. A randomized controlled trial in individuals with spinal cord injury. Spinal Cord Ser Cases. Mar 8, 2024;10(1):10. [doi: ] [Medline: 38459049]

28. Kowalczewski J, Chong SL, Galea M, Prochazka A. In-home tele-rehabilitation improves tetraplegic hand function. Neurorehabil Neural Repair. Jun 2011;25(5):412-422. [doi: ] [Medline: 21372246]

29. Kryger MA, Crytzer TM, Fairman A, et al. The effect of the interactive mobile health and rehabilitation system on health and psychosocial outcomes in spinal cord injury: randomized controlled trial. J Med Internet Res. Aug 28, 2019;21(8):e14305. [doi: ] [Medline: 31464189]

30. Lawrason SVC, Martin Ginis KA. Evaluating the feasibility, acceptability, and engagement of an mHealth physical activity intervention for adults with spinal cord injury who walk: a randomized controlled trial. J Sport Exerc Psychol. Apr 1, 2023;45(2):61-76. [doi: ] [Medline: 36870347]

31. Li J, Li QP, Yang BH. Participatory continuous nursing using the WeChat platform for patients with spinal cord injuries. J Int Med Res. May 2021;49(5):3000605211016145. [doi: ] [Medline: 34038208]

32. Li QP, Li J, Pan HY. Effects of online home nursing care model application on patients with traumatic spinal cord injury. Risk Manag Healthc Policy. 2021;14:1703-1709. [doi: ] [Medline: 33935524]

33. Liu T, Xie S, Wang Y, et al. Effects of app-based transitional care on the self-efficacy and quality of life of patients with spinal cord injury in China: randomized controlled trial. JMIR Mhealth Uhealth. Apr 1, 2021;9(4):e22960. [doi: ] [Medline: 33792555]

34. Liu Y, Hasimu M, Jia M, et al. The effects of APP-based intervention for depression among community-dwelling individuals with spinal cord injury: a randomized controlled trial. Arch Phys Med Rehabil. Feb 2023;104(2):195-202. [doi: ] [Medline: 36332676]

35. Mackelprang JL, Hoffman JM, Garbaccio C, Bombardier CH. Outcomes and lessons learned from a randomized controlled trial to reduce health care utilization during the first year after spinal cord injury rehabilitation: telephone counseling versus usual care. Arch Phys Med Rehabil. Oct 2016;97(10):1793-1796. [doi: ] [Medline: 27039058]

36. Mercier HW, Ni P, Houlihan BV, Jette AM. Differential Impact and Use of a Telehealth Intervention by Persons with MS or SCI. Am J Phys Med Rehabil. Nov 2015;94(11):987-999. [doi: ] [Medline: 25888652]

37. Migliorini C, Sinclair A, Brown D, Tonge B, New P. A randomised control trial of an Internet-based cognitive behaviour treatment for mood disorder in adults with chronic spinal cord injury. Spinal Cord. Sep 2016;54(9):695-701. [doi: ] [Medline: 26690861]

38. Pain H, Soopramanien A, Dallolio L, et al. Outcomes from a randomized controlled trial of telerehabilitation for people with spinal cord injuries. J Telemed Telecare. Jul 2007;13(1_suppl):46-48. [doi: ]

39. Rintala DH, Garber SL, Friedman JD, Holmes SA. Preventing recurrent pressure ulcers in veterans with spinal cord injury: impact of a structured education and follow-up intervention. Arch Phys Med Rehabil. Aug 2008;89(8):1429-1441. [doi: ] [Medline: 18674978]

40. Swarnakar R, Yadav S, Wadhwa S, Venkataraman S. Effectiveness of telerehabilitation in persons with spinal cord injury during the COVID-19 pandemic (TELE-SCOPE): a single-center, double-blind, randomized controlled trial. Cureus. Jul 2023;15(7):e41513. [doi: ] [Medline: 37551233]

41. Worobey LA, Rigot SK, Hogaboom NS, Venus C, Boninger ML. Investigating the efficacy of web-based transfer training on independent wheelchair transfers through randomized controlled trials. Arch Phys Med Rehabil. Jan 2018;99(1):9-16. [doi: ] [Medline: 28782541]

42. Jaglal SB, Allin SJ, Craven BC, et al. A pilot randomised controlled trial of the Spinal Cord Injury and You (SCI&U) online peer health coaching self-management program. Pilot Feasibility Stud. Jan 28, 2026;12(1):29. [doi: ] [Medline: 41593819]

43. Young HJ, Mohanraj S, Malone LA, et al. Feasibility, Usability, and Acceptability of a Randomized Controlled Trial Evaluating Teleexercise Interventions for Individuals with Spinal Cord Injury: Interim Analysis of the Spinal Cord Injury Program in Exercise (SCIPE) Study. Arch Rehabil Res Clin Transl. Sep 2025;7(3):100495. [doi: ] [Medline: 40980516]
